# Merging Arcs to Produce Acyclic Phylogenetic Networks and Normal Networks

**DOI:** 10.1007/s11538-021-00986-1

**Published:** 2022-01-04

**Authors:** Stephen J. Willson

**Affiliations:** Department of Mathematics, Iowa State University, Ames, IA 50011 USA

**Keywords:** Phylogeny, Network, Phylogenetic network, CSD map, Normal network

## Abstract

As phylogenetic networks grow increasingly complicated, systematic methods for simplifying them to reveal properties will become more useful. This paper considers how to modify acyclic phylogenetic networks into other acyclic networks by contracting specific arcs that include a set *D*. The networks need not be binary, so vertices in the networks may have more than two parents and/or more than two children. In general, in order to make the resulting network acyclic, additional arcs not in *D* must also be contracted. This paper shows how to choose *D* so that the resulting acyclic network is “pre-normal”. As a result, removal of all redundant arcs yields a normal network. The set *D* can be selected based only on the geometry of the network, giving a well-defined normal phylogenetic network depending only on the given network. There are CSD maps relating most of the networks. The resulting network can be visualized as a “wired lift” in the original network, which appears as the original network with each arc drawn in one of three ways.

## Introduction

A phylogenetic tree is a directed tree whose vertices represent biological species, whose leaves typically correspond to known extant species, and whose branchings indicate speciation events, usually by genetic mutation. As such, internal vertices have in-degree one and out-degree at least two (except for the root with in-degree zero). In the last decades it has become clear that other events such as hybridization and lateral gene transfer are also important in evolution, even though they are not easily modeled using phylogenetic trees (Delwiche and Palmer 1996; Doolittle and Bapteste 2007; Inagaki et al. 2002; Jones et al. 2013). As a result there is interest in phylogenetic networks, in which some vertices have in-degree two or higher, corresponding to such events (Moret et al. 2004; Solís-Lemus et al. 2016). Overviews of phylogenetic networks may be found in Steel (2016) and Huson et al. (2010).

A phylogenetic *X*-network is an acyclic directed graph in which the leaves are identified with a particular collection *X* of species, usually extant species. We assume that a phylogenetic *X*-network describes gene flow, and each vertex corresponds to a biological species. Such phylogenetic networks can be quite complicated. The focus will be on simplifying such networks by recursively merging the ends of particular arcs in a natural manner. We will then apply the results to study simplification into a normal network.

In this paper, an *X*-network is a directed graph in which the leaves are identified with members of a particular set *X*. Our notion of an *X*-network is broad. Vertices can have in-degree and/or out-degree greater than two, so we are not assuming that the networks are binary. An exact definition is given in Sect. 2, along with other basic notions. In Sect. 3 we describe constructions which do not necessarily yield acyclic networks, and then find conditions that ensure that the results are acyclic. Hence in this paper an *X*-network need not be acyclic, and we will refer to one that is acyclic as an *acyclic X-network.* In turn, an acyclic *X*-network that has no vertices with both out-degree and in-degree equal to one is a *phylogenetic* *X*-network.

One measure of the complexity of an acyclic *X*-network is the number of vertices. In terms of bounds on the number of vertices we have the following comparisons between certain families of networks. The definitions of these families are given in Sect. 2. (The result is from Willson (2010) with slight changes since, in Willson (2010), *X* contained the root as well as the leaves.)

### Theorem 1.1

Suppose $N = (V,A,\rho ,\phi )$ is an acyclic *X*-network and $n = |X|$, $v = |V|$. Assume $n\ge 2$ and there are no vertices of out-degree one.

(a) If *N* is a rooted tree, then $v \le 2n-1$.

(b) If *N* is normal, then $v \le n(n+1)/2$.

(c) If *N* is regular, then $v \le 2^n -1$.

(d) If *N* is tree-child, then *v* is unbounded.

The fact that for normal networks the number of vertices grows at worst quadratically with *n* indicates that normal networks are potentially a more tractable network type than regular or tree-child. Also indicative of their tractable nature is the fact (Steel 2016) that the number of hybrid vertices is at most $n-2$. Yet another indication is that binary normal networks are determined by their caterpillars on three and four leaves (Linz and Semple 2020).

A vertex *v* of the *X*-network $N=(V,A,\rho ,\phi )$ is *visible* (Francis et al. 2021; Huson et al. 2010) if there exists a leaf $\phi (x)$ such that every path in *N* from the root $\rho$ to $\phi (x)$ includes *v*. In a tree-child network, every vertex is visible (Cardona et al. 2009). Since any normal network is tree-child, every vertex of a normal network is visible, yielding another useful property of normal networks.

As in Pardi and Scornavacca (2015) we take the view that rather than try to deal with networks that are possibly not identifiable, it is desirable to focus instead on networks that are sufficiently tractable to be tested with data. Since every vertex of a normal network is visible, potentially every vertex of a normal network can be so tested, and simplification into uniquely determined normal networks will become useful.

This paper relies on results from Willson (2012). This earlier paper focused on networks that were not necessarily acyclic. This current paper extends the results to ensure that the constructed networks are acyclic. If *N* is a given network and *D* is a list of certain arcs in *N* satisfying a weak condition, this paper in Sect. 3 computes the result $M_D(N)$ of merging the arcs in *D* as well as certain additional arcs required to ensure that $M_D(N)$ is acyclic. Of interest will be the choice of *D* so as to obtain ultimately a normal network.

In Sect. 5 we study the result $\text {R}(N)$ of removing all “redundant” arcs from *N*. In Sect. 7 we describe ways to find sets *D* of arcs of *N* such that $\text {R}(M_D(N))$ is a normal network.

Combining these techniques we describe in Sect. 7 a method, given an *X*-network *N*, to construct a normal acyclic *X*-network $\text {Norm}(N)$ which is a phylogenetic *X*-network depending only on the geometry of *N*. The construction makes no arbitrary choices such as between different parents or children.

As phylogenetic *X*-networks grow increasingly complicated, it will become useful to “simplify” them. Simplification into a normal network may make them easier to interpret since normal networks are potentially tractable.

If *N* and *M* are *X*-networks, a connected surjective digraph map (CSD map) $$f:N \rightarrow M$$ is a surjective map $$f: V(N) \rightarrow V(M)$$ with various properties. (See Willson 2012 and Sect. 2 of this paper.) The merging procedure in this paper always yields a CSD map $$\psi : N \rightarrow M_D(N)$$. Results in Willson (2012) show that there is then a “wired lift” of $$M_D(N) $$ into *N*, from which properties of $$M_D(N)$$ can be visualized in *N*. The wired lift is not a subnetwork of *N* in the usual sense.

Section 6 of this paper generalizes the notion of “wired lift”. As a result we obtain a wired lift of $\text {Norm}(N)$ into *N*, even though there is usually no CSD map from *N* to $\text {Norm}(N)$. The wired lift is visualized by drawing the diagram of *N* with each arc drawn in one of three different ways. Thus we can visualize the resulting normal network by looking at a redrawn diagram of *N*. The current author thinks such visualizations can provide a tool for better understanding complicated networks.

Section 8 contains two examples of the methods applied to published networks based on biological data. Section 9 contains some discussion.

Francis et al. (2021) describe an elegant procedure, given an acyclic *X*-network *N*, to find a related, uniquely determined, normal *X*-network, which I will denote $\text {FHS}(N)$. Its calculation is based on locating the visible vertices of *N*. The fast program *PhyloSketch* (Huson and Steel 2020) is available to compute it. The paper (Francis et al. 2021) assumes that non-root vertices have either in-degree one or out-degree one. Nevertheless, visibility of vertices is well-defined for the *X*-networks defined in this paper and their procedure applies to any acyclic *X*-network in our sense. I therefore use $\text {FHS}(N)$ to represent the result of this extension of their method. We will occasionally compare $\text {FHS}(N)$ with $\text {Norm}(N)$.

## Basic Notions

Let $N = (V,A)$ be a directed graph, where *V* is a finite set of vertices and *A* is the set of arcs. An arc (*u*, *v*) is regarded as directed from *u* to *v*, so we call *u* a *parent* of *v* and *v* a *child* of *u*. We assume *N* is a *simple* graph: there are no loops (*u*, *u*); and there is at most one arc (*a*, *b*) for $a\ne b$. We may sometimes denote $V(N)=V$ or $A(N)=A$.

If $N=(V,A)$ is a directed graph, the *corresponding undirected graph* *Und*(*N*) $=(V,E)$ is the graph where $\{u,v\} \in E$ iff either $(u,v)\in A$ or $(v,u)\in A$. Thus, arcs are replaced by *edges* and are not directed. In this paper, *N* will always refer to a directed graph unless otherwise specified.

The *in-degree* of a vertex *v* in *N*, denoted *indeg*(*v*) or *indeg*(*v*; *N*) , is the number of arcs (*u*, *v*), i.e. the number of parents of *v*. The *out-degree* of a vertex *v*, denoted *outdeg*(*v*) , is the number of arcs (*v*, *u*) , i.e., the number of children of *v*.

We shall not assume that our directed graphs are binary. Thus a vertex *v* may have $outdeg(v)> 2$ or $indeg(v)>2$ or both.

A *leaf* is a vertex $x \in V$ with out-degree 0. A *root* is a vertex $\rho \in V$ with in-degree 0. A vertex *v* is *hybrid* or *reticulate* if $indeg(v) \ge 2$. A child *u* of *v* is a *tree-child* if $indeg(u) = 1$, so (*v*, *u*) is the only arc coming into *u*. A vertex *v* is *trivial* if $indeg(v)=outdeg(v) = 1$. A trivial vertex merely subdivides an arc, and we will often systematically suppress trivial vertices.

If *u* and *v* are vertices, a *path* or, for emphasis, a *directed path* from *u* to *v* is a sequence of vertices $$u = u_0,u_1, u_2, \cdots , u_n = v$$ such that for all *i*, $$1 \le i \le n$$, $$ (u_{i-1}, u_i) \in A$$. The *length* of the path is the number *n* of arcs. Note that the arcs are uniquely determined by the vertices in the list since *N* is a simple graph. No two successive vertices can be the same since there are no loops. We say the path *contains* arc $$ (u_k, u_{k+1})$$ for $$k = 0, \cdots , n-1$$. In some situations we may focus on a certain part of the path such as $$u_2,u_3,u_4$$; we may refer to such a portion as a *segment*. (For example, in certain circumstances we might modify the path by replacing a segment $$u_2,u_3,u_4$$ by a segment $$u_2, v_1, v_2, u_4$$.)

The path of length 0 consisting only of $$ u_0$$ is the *trivial* path at $$u_0$$. A path $$u_0, u_1, u_2, \cdots ,u_n$$ is *closed* if $$n>0$$ and $$u_0 = u_n$$. A closed path is a *cycle*.

Let *X* be a nonempty finite set. In the applications, *X* is usually a set of extant biological species. An *X*-*directed graph* *N* is a 4-tuple $(V,A,\rho ,\phi )$ where (*V*, *A*) is a simple directed graph; $\rho$ is a distinguished node; and $\phi$ is a map $\phi : X \rightarrow V$.

An *X*-*network* *N* is an *X*-directed graph $(V,A, \rho , \phi )$ such that

(N1) *V* is a finite set (the set of *nodes* or *vertices*).

(N2) *A* (the set of *arcs*) is a finite set of ordered pairs (*u*, *v*) with *u* and *v* distinct members of *V*.

(N3) $\rho$, called the *root*, is a node with in-degree 0.

(N4) The map $\phi : X \rightarrow V$ is one-to-one.

(N5) Each leaf is a vertex with in-degree 1 and hence has a unique parent.

(N6) The image of $\phi$ is the set of leaves.

(N7) $\rho$ is the only vertex with in-degree 0.

(N8) For each $v \in V$ there is a path from $\rho$ to *v*.

(N9) For each $v \in V$ there is a path from *v* to some leaf.

An *acyclic* *X*-network is an *X*-network that also satisfies

(N10) *N* has no cycles.

Following Steel (2016), we define a *phylogenetic* *X*-network to be an acyclic *X*-network that contains no trivial vertices.

These assumptions are not intended to be the minimal possible; rather, they tell the properties we will utilize the most.

If $x\in X$ the unique parent of $\phi (x)$ by (N5) will be denoted *p*(*x*) or *p*(*x*; *N*). The arc of form $(p(x),\phi (x))$ for some $x\in X$ will be called the *x-arc*. If *x* is not specified, any such arc will be called an *X-arc*.

Suppose *N* is an *X*-network. By (N4) and (N6) we may identify *X* with the set of leaves.

If there is a directed path from *u* to *v* then we write $u \le v$. The trivial path shows $u\le u$ for all $u \in V$. (N9) says for each $v \in V$ there is $x \in X$ with $v \le \phi (x)$. (N8) says that for any $v \in V$, $\rho \le v$. If the *X*-network is acyclic, then $\le$ is a partial order; otherwise it is possible that for distinct vertices *u* and *v* we have $u\le v\le u$.

Two *X*-networks or two *X*-directed graphs $N = (V, A,\rho ,\phi )$ and $N' = (V', A' ,\rho ',\phi ')$ are *X*-*isomorphic* iff there exists a map $f: V \rightarrow V'$ such that

(i) *f* is one-to-one and surjective.

(ii) $$f (\rho ) = \rho '$$.

(iii) $f \circ \phi = \phi '$ (thus $f(x) = x$ for $x \in X$, with the obvious interpretations).

(iv) $$ (a,b) \in A$$ iff $$ (f(a), f(b)) \in A'$$.

In this situation, *N* and $N'$ are essentially the same and we write $N \cong N'$.

For each $v \in V$, we write *cl*(*v*; *N*) (or *cl*(*v*) when *N* is understood) for $cl(v;N) = \{x \in X: v \le \phi (x)\}$. We call it the *cluster* of *v*. Note that $cl(\rho ) = X$ by (N8). By (N9) for every vertex *v*, *cl*(*v*) is nonempty.

It is immediate that if $u \le v$ then $cl(v) \subseteq cl(u)$.

Let $Cl(N) = \{cl(v): v \in V\}$ be the set of clusters of *N*.

Let *N* be an *X*-labeled graph. An arc (*a*, *b*) is *redundant* or a *short-cut* if there exists a path $a = u_0, u_1, \cdots , u_n = b$, $n\ge 2$, that does not contain the arc (*a*, *b*). Thus, there is no $k\le n-1$ such that $u_k=a$ and $u_{k+1} = b$. Such a path is called a *lengthening* or a *lengthening path* of (*a*, *b*). Examples will be seen in several figures later, such as Fig. 3. If $x\in X$ then the arc $(p(x),\phi (x))$ cannot be redundant since any such lengthening path would have to satisfy $u_{n-1}=p(x)$ by (N5).

We shall have need of the following result:

### Theorem 2.1

Suppose *N* is an acyclic *X*-network. Suppose there is a directed path in *N* from *a* to *b*. Then, a directed path in *N* from *a* to *b* of maximal length contains no redundant arc.

### Proof

Since the vertex set is finite and there are no cycles, there is an upper bound to the length of a path. Suppose $a = u_0, u_1, \cdots , u_k = b$ is a directed path *P* in *N* of maximal length *k*. If the result is false, we may assume that for some $i<k$, $(u_i, u_{i+1})$ is redundant. In that case there is a directed path $u_i = w_0, w_1, \cdots , w_j = u_{i+1}$ with $j \ge 2$. We can then lengthen the path *P* by replacing the segment $u_i, u_{i+1}$ by $w_0, \cdots , w_j$, a contradiction. $\square$

There are several types of *X*-networks which will be of interest:

An acyclic *X*-network *N* is *tree-child* (Cardona et al. 2009) if every vertex that is not a leaf has a tree-child.

An acyclic *X*-network $N = (V,A,\rho ,\phi )$ (possibly not satisfying (N5)) is *regular* (Baroni et al. 2004) if

(1) the cluster map $cl: V \rightarrow P(X)$ is one-to-one, where *P*(*X*) is the power set of *X*;

(2) *N* has no redundant arcs; and

(3) $$u \le v$$ iff $$cl(v) \subseteq cl(u)$$.

An acyclic *X*-network *N* is *normal* (Willson 2010) if

(1) *N* is tree-child; and

(2) *N* contains no redundant arc.

Sometimes there are small differences in the definition of a network. In Baroni et al. (2004) and Willson (2010) the authors do not assume condition (N5). In Baroni et al. (2004) no vertex can have out-degree one. Particularly simple are normal networks in which no vertex has out-degree one, since these are regular (Willson 2010).

Let *N* and $N'$ be acyclic *X*-networks. One interesting way to compare them is their *Robinson-Foulds distance* $d_{RF}(N,N')$ defined as the number of members of *Cl*(*N*) and $Cl(N')$ which are present in one but not both (an extension of Robinson and Foulds (1981) for trees). It is symmetric and satisfies the triangle inequality. For certain classes of *X*-networks $d_{RF}$ is a metric. As an example, for fixed *X*, it is a metric on the collection of regular *X*-networks (Baroni et al. 2004).

If *N* is a normal *X*-network, let *S*(*N*) denote the result of contracting every arc (*u*, *v*) such that $outdeg(u)=1$. For example, suppose in *N*, for some $x\in X$, *p*(*x*) is hybrid and has out-degree one. Then in *S*(*N*) the arc $(p(x),\phi (x))$ in *N* will have been contracted, and *S*(*N*) will not satisfy (N5). Thus in *S*(*N*) a leaf can be hybrid. Moreover, any trivial vertices will have been suppressed.

The following result shows that two normal networks $N_1$ and $N_2$ such that $d_{RF}(N_1,N_2)=0$ are essentially the same.

### Lemma 2.2

(1) If *N* is a normal *X*-network, then *S*(*N*) is a regular *X*-network and $Cl(S(N))=Cl(N)$.

(2) Suppose $N_1$ and $N_2$ are normal *X*-networks and $d_{RF}(N_1,N_2)=0$. Then $S(N_1)\cong S(N_2)$.

### Proof

(1) For any *X*-network *N*, if (*u*, *v*) is an arc and $outdeg(u)=1$, it is immediate that $cl(u)=cl(v)$. Hence $Cl(S(N))=Cl(N)$. Moreover, if *N* is normal then *S*(*N*) remains normal and hence is a regular network (Willson 2010).

(2) If $d_{RF}(N_1,N_2)=0$, then $Cl(N_1)=Cl(N_2)$. Hence $Cl(S(N_1))=Cl(N_1)=Cl(N_2)=Cl(S(N_2))$, so $d_{RF}(S(N_1), S(N_2))=0$. The result follows from the fact (Baroni et al. 2004) that $d_{RF}$ is a metric on regular *X*-networks. $\square$

The $d_{RF}$ distance has the interesting property that since it is defined for all acyclic *X*-networks, it can be used to compare how well various networks of various types “approximate” a given network. For example, if *N* is a complicated acyclic *X*-network and *T* and $T'$ are *X*-networks that are rooted trees, then *T* might be a better approximation to *N* than $T'$ if $d_{RF}(N,T) < d_{RF}(N,T')$.

In this paper we will be “simplifying” an acyclic *X*-network *N* into a normal *X*-network $N'$. From this point of view we would prefer that $d_{RF}(N,N')$ is as small as possible.

Let $N = (V,A,\rho ,\phi )$ and $N' = (V',A', \rho ', \phi ')$ be *X*-directed graphs. A *connected surjective digraph* (CSD) map (Willson 2012) $\psi : N \rightarrow N'$ is a map $\psi : V \rightarrow V'$ such that

(C1) $$\psi $$ is surjective.

(C2) For each arc $(u,v) \in A$, either $\psi (u) = \psi (v)$ or else $(\psi (u), \psi (v)) \in A'$. In the latter case we may write $\psi (u,v)=(\psi (u),\psi (v))$. (Thus $\psi$ is a *digraph map*).

(C3) For each $x \in X$, $\psi ( \phi (x)) = \phi '(x)$. More simply, $\psi (x) = x$.

(C4) $$\psi (\rho ) = \rho '$$.

(C5) For each $(u',v') \in A'$ there exists *u*, *v* in *V* such that $\psi (u) = u'$, $\psi (v)=v'$, and $(u,v) \in A$.

(C6) For each $v' \in V'$, $\psi ^{-1}(v')$ consists of the vertices of a connected subgraph of *N*. Thus in the undirected graph *Und*(*N*) of *N*, if $W = \psi ^{-1}(v')$, the induced subgraph with vertex set *W* and edge set $\{\{u,v\}: \psi (u)=\psi (v)=v', (u,v) \in A$ or $(v,u) \in A\}$ is connected.

Note that if $u\le v$ in *N* and $\psi :N \rightarrow N'$ is a CSD map, then $\psi (u) \le \psi (v)$ in $N'$.

Let $N = (V,A,\rho ,\phi )$ and $N' = (V',A', \rho ', \phi ')$ be *X*-networks. A CSD-map $\psi : N \rightarrow N'$ is *leaf-preserving* if for each $x\in X$

(C7) $u=\phi (x)$ is the only vertex in *V* such that $\psi (u)=\phi '(x)$; thus $\psi ^{-1}(\phi '(x))=\{\phi (x)\}$; and

(C8) the *x*-arc $(p(x),\phi (x))\in A$ is taken to the *x*-arc $(\psi (p(x)),\phi '(x))$; thus $\psi (p(x;N))=p(x;N')$.

If $\psi _1: N \rightarrow N'$ and $\psi _2: N' \rightarrow N''$ are CSD maps, then it is proved in Willson (2012) that the composition $\psi = \psi _2 \circ \psi _1: N \rightarrow N''$ is also a CSD map. If both maps are leaf-preserving, then it is easy to see that the composition is also leaf-preserving. We will use this fact repeatedly.

Note that in Willson (2012) the term “*X*-network” refers to what in this paper is an *X*-directed graph satisfying (N1), (N2), (N3), (N4), (N6), (N7), (N8), and (N9). Thus the networks in Willson (2012) were not required to be acyclic. Of interest in this current paper is the behavior when the final networks are required to be acyclic, as are phylogenetic networks in biology. The CSD maps $\phi :N\rightarrow N'$ become more useful to biologists when both *N* and $N'$ are required to be acyclic.

## Contraction of Arcs

Here is a summary of this fundamental section: The basic tool used in this paper is that of successively contracting arcs in an *X*-network. Suppose *N* is an *X*-network and *D* is a subset of its arcs. In this section under weak conditions we describe how to construct an *X*-network $Q_D(N)$ by merging just the arcs of *D*. In general $Q_D(N)$ may contain cycles. When *D* is “strongly closed” we show that $Q_D(N)$ is acyclic. Moreover, any *D* has a unique “strong closure” *K*(*D*) which contains *D* and is strongly closed. Hence, we are able to define $M_D(N)=Q_{K(D)}(N)$ as a uniquely determined acyclic *X*-network that results from contracting the arcs of *D* and also the other arcs needed for acyclicity. The sections after this one will rely on the iterated use of this construction. The fundamental problem studied in this paper is, roughly, how to choose *D* so that we can find a normal network from $M_D(N)$.

Let $N=(V,A,\rho ,\phi )$ be an *X*-network. Suppose $\sim$ is an equivalence relation on *V*. Let [*v*] denote the equivalence class of $v\in V$. Let $P(V,\sim )$ denote the set of equivalence classes of *V* under $\sim$.

As in Willson (2012) the *quotient digraph* $N'=(V',A', \rho ', \phi ')$ is defined by

(1) $$V' = P(V,\sim )$$.

(2) $$\rho '=[\rho ]$$.

(3) For each $$x\in X$$, $$\phi '(x)=[\phi (x)]$$.

(4) Let [*u*] and [*v*] be in $V'$. There is an arc $([u],[v]) \in A'$ if and only if $[u]\ne [v]$ and there exist $u_0\in [u]$ and $v_0\in [v]$ such that $(u_0,v_0)\in A$.

We will denote this quotient digraph by $P(N,\sim )$ or $N/\sim$. Note that by (4), $P(N,\sim )$ contains no loops and is a simple graph.

The equivalence relation $\sim$ is *connected* if each equivalence class [*v*] is connected in *N*. An equivalence class [*v*] is *convex* if, whenever $u_0, u_1, \cdots , u_k$ is a path in *N* with both $u_0\in [v]$ and $u_k\in [v]$, then for all *i*, $0\le i\le k$, $u_i\in [v]$. We say that $\sim$ is *convex* if each equivalence class [*v*] is convex.

The relation $\sim$ is *root-preserving* if the equivalence class $[\rho ]$ is convex. The relation $\sim$ is *leaf-preserving* provided

(1) if $x,y\in X$ and $x\ne y$, then $[\phi (x)]\ne [\phi (y)]$; and

(2) for any $x\in X$, $[\phi (x)] = \{\phi (x)\}$. Thus $\phi (x)$ is the only vertex *u* such that $u\sim \phi (x)$.

Let $N = (V,A,\rho ,\phi )$ and $N' = (V',A', \rho ', \phi ')$ be *X*-networks. A CSD-map $\psi : N \rightarrow N'$ is *leaf-preserving* if for each $x\in X$

(1) $u=\phi (x)$ is the only vertex in *V* such that $\psi (u)=\phi '(x)$; thus $\psi ^{-1}(\phi '(x))=\{\phi (x)\}$; and

(2) the *x*-arc $(p(x),\phi (x))\in A$ is taken to the *x*-arc $(\psi (p(x)),\phi '(x))$; thus $\psi (p(x;N))=p(x;N')$.

The following result is similar to Theorem 3.1 of Willson (2012). We outline the proof here again because some of the definitions have slightly changed, for example, to allow for (N5) and leaf-preserving CSD maps.

### Theorem 3.1

Let $N=(V,A,\rho ,\phi )$ be an *X*-network. Let $\sim$ be a connected leaf-preserving and root-preserving equivalence relation on *V*, and let $N'=(V',A',\rho ,\phi ')$ be the quotient digraph $N/\sim$. Then

(1) $N'$ is an *X*-network.

(2) The natural map $\psi :V\rightarrow V'$ given by $\psi (v)=[v]$ induces a leaf-preserving CSD map $\psi :N\rightarrow N'$.

### Proof

First we prove (1). (N1) and (N2) are immediate. (N4) is immediate since $\sim$ is leaf-preserving. If $(u,v)\in A$, then it is immediate that either $[u]=[v]$ or else $([u],[v])\in A'$. It follows that if there is a path from *a* to *b* in *N*, then there is a path from $\psi (a)$ to $\psi (b)$ in $N'$. Thus (N8) is true.

Each $[\phi (x)]$ for $x\in X$ is a leaf of $N'$ since otherwise there would be an arc $([\phi (x)],[u])$ for some [*u*] and hence an arc from some $v\in [\phi (x)]$ to $u_0\in [u]$. But since $\sim$ is leaf-preserving, $v=\phi (x)$ and so $(\phi (x),u_0)\in A$, contradicting that $\phi (x)$ is a leaf of *N*.

Let $[v]\in V'$. By (N9) and (N6) for *N* there is a path in *N* from *v* to $\phi (x)$ for some $x\in X$. Then, there is a path from [*v*] to $[\phi (x)]$ in $N'$, proving (N9) for $N'$.

Suppose [*v*] is a leaf of $N'$. If *v* is not a leaf of *N*, then there is a path in *N* from *v* to $\phi (x)$ for some $x\in X$ by (N9) and (N6). Let the path be $v=u_0, u_1, \cdots , u_k =\phi (x)$ with $k\ge 1$. Since [*v*] is a leaf, it follows $[v]=[u_1]$ since otherwise $([v],[u_1])\in A'$. If $k=1$ then $[v]=[\phi (x)]$ so $v=\phi (x)$ because $\sim$ is leaf-preserving. If $k>1$, $[u_1]=[u_2]$ since otherwise $([u_1],[u_2])\in A'$ while $[u_1]=[v]$ is a leaf. It follows by an easy inductive argument that $[v]=[\phi (x)]$. Thus, every leaf of $N'$ has the form $\phi '(x)$ for some $x\in X$, proving (N6).

Suppose in $N'$ there is an arc $([u],[\rho ])$. Then, there exists $u_0\in [u]$ and $v_0\in [\rho ]$ such that there is an arc $(u_0,v_0)$. By (N8) there is a path in *N* from $\rho$ to $u_0$ and from there to $v_0$ via the arc $(u_0,v_0)$. This path from $\rho$ to $v_0$ satisfies that $[\rho ]=[v_0]$, so, since $\sim$ is root-preserving, it follows that each vertex in the path lies in $[\rho ]$. In particular $u_0\in [\rho ]$ so $[u]=[u_0]=[\rho ]$, contradicting the arc $([u],[\rho ])$. This proves (N3).

For (N7), suppose [*u*] has in-degree 0. By (N8) there is a path from $\rho$ to *u*, say $\rho =u_0, u_1, \cdots , u_k = u$. If $k\ge 1$ then $(u_{k-1},u)\in A$. Since [*u*] has in-degree 0, it follows that $u_{k-1}\in [u]$ and so $[u_{k-1}]=[u]$. If $k-1\ge 1$ then $(u_{k-2},u_{k-1})\in A$; since [*u*] has in-degree 0, it follows $u_{k-2}\in [u]$, so $[u_{k-2}]=[u]$. Repeating this argument we see by induction that $[u_0]=[u]$. But $[u_0] =[\rho ]$, whence $[u]=[\rho ]$, proving (N7).

For (N5), we know from (N6) above that each leaf of $N'$ is of form $[\phi (x)]$. By (N5) for *N*, $\phi (x)$ has a unique parent in *N*, denoted *p*(*x*), so $(p(x),\phi (x))\in A$. By the definition of $A'$ either $[p(x)] = [\phi (x)]$ or $([p(x)],[\phi (x)])\in A'$. Since $\sim$ is leaf-preserving, the former is not possible, so $([p(x)],[\phi (x)])\in A'$. Suppose $[u]\ne [p(x)]$ and $([u],[\phi (x)])\in A'$. Since $[\phi (x)]=\{\phi (x)\}$ because $\sim$ is leaf-preserving, we may assume $(u,\phi (x))\in A$. This implies $u=p(x)$ by (N5) in *N*, proving (N5) for $N'$.

This completes the proof of (1).

We now prove (2). (C1) is immediate since every vertex of $N'$ has the form [*v*]. (C2) is immediate since if $(u,v)\in A$ and $[u]\ne [v]$, then by definition $([u],[v])\in A'$. (C3), (C4), and (C5) are immediate from the definition of $A'$. For (C6) note that for any $[v]\in v'$, $\psi ^{-1}([v])=[v]$, the latter as a set. Since $\sim$ is connected, [*v*] is connected as well.

(C7) restates that $[\phi (x)] = \{\phi (x)\}$, which is true since $\sim$ is leaf-preserving. (C8) restates the fact, proved above, that $([p(x)], [\phi (x)])$ is the unique arc entering $[\phi (x)].$ This proves $\psi$ is leaf-preserving, completing the proof of (2). $\square$

We do not claim that $N/\sim$ is acyclic, even if *N* is acyclic.

We will refer to the map $\psi$ as the *projection* from *N* to $N/\sim$.

Let $N=(V,A,\rho ,\phi )$ be an *X*-network. Let *D* be a subset of *A*. Define a relation $\sim _D$ on *V* by saying vertices *a* and *b* satisfy $a\sim _D b$ iff there is a sequence (not necessarily a path) $a = u_0, u_1, \cdots , u_k = b$ such that for $0\le i\le k-1$, either $(u_i,u_{i+1}) \in D$ or $(u_{i+1},u_i) \in D$.

Let *D* be a subset of *A*. A path $u_0, \cdots , u_k$ is called a *D-path* provided that for each *i*, $0\le i \le k-1$, $(u_i,u_{i+1})\in D$. Call *D* *root-preserving* if whenever $\rho =u_0, \cdots , u_k=a$ is a *D*-path, then every path from $\rho$ to *a* is a *D*-path.

### Theorem 3.2

Let $N=(V,A,\rho ,\phi )$ be an *X*-network. Let *D* be a subset of *A*. Then

(1) $\sim _D$ is a connected equivalence relation.

(2) $\sim _D$ is leaf-preserving if *D* contains no *X*-arc.

(3) $\sim _D$ is root-preserving if *D* is root-preserving.

### Proof

(1) For any vertex *v* the trivial path at *v* shows $v\sim _D v$. Suppose $a\sim _D b$ because of the path $a = u_0, u_1, \cdots , u_k = b$ such that for $0\le i\le k-1$, either $(u_i,u_{i+1}) \in D$ or $(u_{i+1},u_i) \in D$. Then $b\sim _D a$ because of the path $b = u_k, u_{k-1}, \cdots , u_0=a$. Transitivity is immediate. Since *D* is a subset of *A*, it is clear that each equivalence class is connected. This proves (1).

Write $[v]_D$ for the equivalence class of *v* under $\sim _D$.

(2) If *D* contains no *X*-arc, then for each $x\in X$, $[\phi (x)]_D = \{\phi (x)\}$. If $x, y \in X$ then $\phi (x)\ne \phi (y)$ by (N4). This proves (2).

(3) Assume that *D* is root-preserving. We must show that the equivalence class $[\rho ]_D$ is convex. Let $u_0, u_1, \cdots , u_k$ be a path in *N* such that $u_0\sim _D \rho$ and $u_k\sim _D \rho$. We must show that each $u_i\sim _D \rho$. Choose a path $\rho =v_0, v_1, \cdots , v_m=u_0$ from $\rho$ to $u_0$; such exists by (N8). Then $v_0, \cdots v_m, u_1, \cdots , u_k$ is a path from $\rho$ to $u_k$. Since $\rho \sim _D u_k$, each vertex $u_i$ satisfies $u_i\sim \rho$, proving (3). $\square$

Let $[v]_D$ (or [*v*] if *D* is understood) denote the equivalence class of *v* under $\sim _D$. We call $\sim _D$ the *equivalence relation determined by D*. It is clearly the smallest equivalence relation $\sim$ (i.e., with the fewest pairs (*u*, *v*) satisfying $u\sim v$), such that, for each arc $(a,b)\in D$, $a\sim b$.

### Theorem 3.3

Let $N=(V,A,\rho ,\phi )$ be an *X*-network. Let *D* be a subset of *A*. Assume *D* contains no *X*-arc and *D* is root-preserving. Then the quotient digraph $N/\sim _D$ is an *X*-network. Moreover, the projection $\psi :N\rightarrow N/\sim _D$ is a leaf-preserving CSD map.

### Proof

By Theorem 3.2, $\sim _D$ is a connected leaf-preserving and root-preserving equivalence relation. The conclusions follow from Theorem 3.1. $\square$

Henceforth if *D* contains no *X*-arc and is root-preserving, we will write $Q_D(N)$ for $N/\sim _D$. We may call it the *quotient X-network* of *N* under *D* and refer to its formation as *contracting* or *merging* the arcs of *D*. In general, $Q_D(N)$ may contain cycles even if *N* is acyclic.

It is not hard to program a computer to find $Q_D(N)$.

**Fig. 1:**
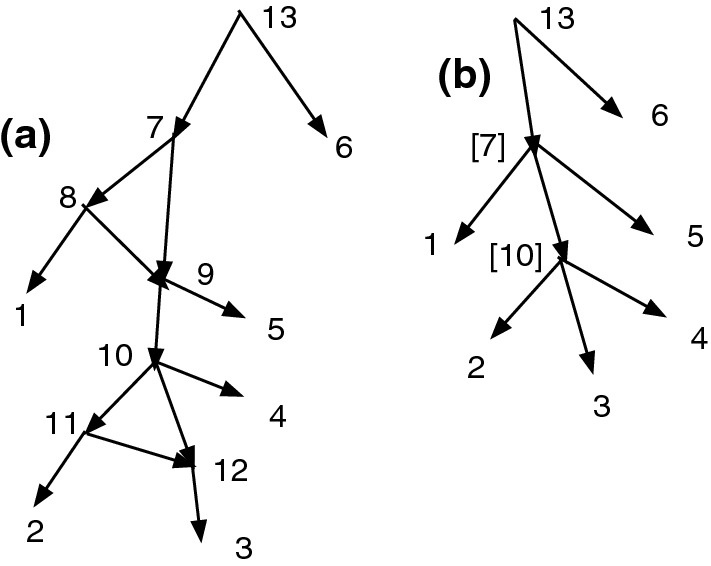
**a** An acyclic *X*-network *N*. Let $D=\{(7,8), (8,9), (10,11), (11, 12)\}$. **b** $Q_D(N)$. Note that *D* is not closed

Figure 1a shows an acyclic *X*-network *N*. Let $D=\{(7,8), (8,9), (10,11), (11, 12)\}$. Write $\sim$ for $\sim _D$, so $7\sim 8\sim 9$ and $10\sim 11\sim 12$. Note that *D* contains no *X*-arc. Note the root $\rho =13$ and $[\rho ]=\{\rho \}$ is convex. Then $Q_D(N)$ is defined and shown in (b). Note $[7]=\{7,8,9\}$ and $[10]=\{10,11,12\}$.

Let $N=(V,A,\rho ,\phi )$ be an *X*-network. A subset $K\subseteq A$ of arcs is *closed* if it satisfies the following: Suppose $a\sim _K b$. Then for every path $a = v_0, v_1, \cdots , v_j = b$ with length $j\ge 1$, for each *i* satisfying $0\le i\le j-1$, $(v_i,v_{i+1}) \in K$. In particular if (*a*, *b*) is an arc and $a\sim _K b$, then $(a,b)\in K$.

In Fig. 1, *D* is not closed since $7\sim _D 9$ yet $(7,9)\notin D$.

### Theorem 3.4

Let $N=(V,A,\rho ,\phi )$ be an *X*-network. Let $D\subseteq A$ be a subset of arcs. If *D* is closed then each equivalence class of $\sim _D$ is convex.

### Proof

Suppose *D* is closed. Let $v\in V$. We wish to prove that [*v*] is convex. Write $\sim$ for $\sim _D$. Let $u_0, u_1, \cdots , u_k$ be a path in *N* where $u_0\sim v$ and $u_k\sim v$. By transitivity, $u_0\sim u_k$. By closure, each arc $(u_i, u_{i+1})\in D$, proving convexity.

Figure 1 shows that the converse of Theorem 3.4 is false. $Q_D(N)$ is acyclic and $\sim _D$ is convex even though *D* is not closed.

Figure 1 also illustrates the fact that often when an arc (*u*, *v*) is merged, the number of vertices drops by one, reducing the resolution. In Fig. 1, *D* contained 4 arcs, and the number of vertices dropped from 13 in (a) to 9 in (b). On the other hand, if $D'=D \cup \{(7,9)\}$, then $Q_{D'}(N) = Q_D(N)$ and the merging of (7,9) does not further reduce the number of vertices.

Let $N=(V,A,\rho ,\phi )$ be an *X*-network. A subset $K\subseteq A$ of arcs is *strongly closed* if it satisfies the following: Suppose there are vertices $a, u_0, u_1, \cdots , u_{2m}=b$ in *V* with $m \ge 1$ such that $a \sim _K u_0$, $(u_0,u_1)\in A$, $u_1\sim _K u_2$, $(u_2,u_3)\in A$, $u_3\sim _K u_4$, $\cdots$, $(u_{2m-2},u_{2m-1})\in A$, $u_{2m-1}\sim _K u_{2m}=b$, and in addition $a\sim _K b$. Then for *k* such that $0\le k\le m-1$ each of the arcs $(u_{2k},u_{2k+1})$ lies in *K*.

### Theorem 3.5

Let $N=(V,A,\rho ,\phi )$ be an *X*-network. Let $D\subseteq A$ be a subset of arcs. If *D* is strongly closed, then *D* is closed. Hence *D* is root-preserving.

### Proof

Suppose *D* is strongly closed. Suppose there is a sequence (not necessarily a path) $a = u_0, u_1, \cdots , u_k = b$ such that for $0\le i\le k-1$, either $(u_i,u_{i+1}) \in D$ or $(u_{i+1},u_i) \in D$. Let $a = v_0, v_1, \cdots , v_j = b$ be a path from *a* to *b*. Then $a \sim _D b$. Trivially, we have $a\sim _D v_0$, $(v_0,v_1)\in A$, $v_1\sim _D v_1$, $(v_1,v_2)\in A$, $v_2\sim _D v_2$, $\cdots$, $(v_{k-1},v_k)\in A$, $v_k\sim _D b$. Since *D* is strongly closed, for $0\le j \le k-1$, the arc $(v_j,v_{j+1})\in D$. Hence *D* is closed.

By Theorem 3.4 it follows that each equivalence class of $\sim _D$ is convex. In particular $[\rho ]$ is convex, so *D* is root-preserving. $\square$

**Fig. 2:**
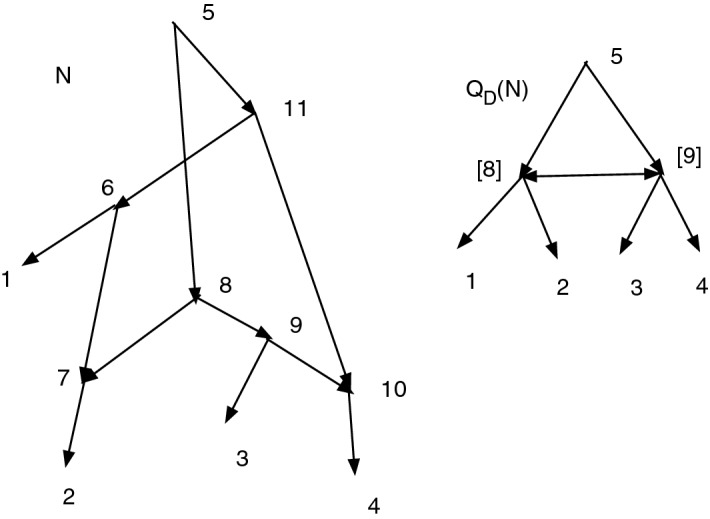
A network *N*. Suppose $D=\{(6,7), (8,7), (9,10), (11,10)\}$. Then *D* is closed but not strongly closed. $Q_D(N)$ has both arcs ([8], [9]) and ([9], [8])

Figure 2 shows a set *D* that is closed but not strongly closed. Indeed, $11\sim _D 11$, $(11,6)\in A$, $6\sim _D 8$, $(8,9)\in A$, $9\sim _D 11$ yet $(11,6)\notin D$ and $(8,9)\notin D$. Note that $Q_D(N)$ contains a cycle since there are both arcs ([8], [9]) and ([9], [8]), where $[8]=\{6,7,8\}$ and $[9]=\{9,10,11\}$.

The next result shows that, if *D* is strongly closed, then $Q_D(N)$ is acyclic.

### Theorem 3.6

Suppose $N=(V,A,\rho ,\phi )$ is an *X*-network. Assume $D\subseteq A$ contains no *X*-arc. If *D* is strongly closed, then $Q_D(N)$ is acyclic.

### Proof

Write $\sim$ for $\sim _D$. Assume *D* is strongly closed. By Theorem 3.5, *D* is root-preserving. By Theorem 3.3, $Q_D(N)$ is an *X*-network. Suppose $Q_D(N)$ contains a cycle hence a path $[a] = [u_0], [u_1], \cdots , [u_k] = [a]$ with $k\ge 2$. Let $\psi :N\rightarrow Q_D(N)$ be the projection CSD map. Since $\psi$ is a CSD map, for each arc $([u_i],[u_{i+1}])$ with $0\le i\le k-1$ there is an arc $(v_i,w_{i+1})\in A$ with $v_i\in [u_i]$ and $w_{i+1}\in [u_{i+1}]$. Thus $a, v_0, w_1,v_1, w_2, \cdots , v_{k-1}, w_k, b$ satisfies $[a]=[v_0]$, $(v_0,w_1)\in A$, $[w_1]=[v_1]$, $(v_1,w_2)\in A$, $[w_2]=[v_2]$, $\cdots$, $[w_{k-1}]=[v_{k-1}]$, $(v_{k-1}, w_k)\in A$, and $[w_k]=[a]$. Thus $a\sim v_0$, $(v_0,w_1)\in A$, $w_1\sim v_1$, $(v_1,w_2)\in A$, $w_2\sim v_2$, $\cdots$, $w_{k-1}\sim v_{k-1}$, $(v_{k-1}, w_k)\in A$, and $w_k\sim a$. Since *D* is strongly closed, each arc $(v_i,w_{i+1})\in D$. Hence $a\sim v_0 \sim w_1 \sim v_1$ $\sim w_2 \cdots$ $\sim v_{k-1} \sim w_k\sim a$. Thus, all the points on the cycle were the same, a contradiction. $\square$

The following theorem shows that from a given *D* we can construct a uniquely determined strongly closed set *K* that contains *D*.

### Theorem 3.7

Let $N=(V,A,\rho ,\phi )$ be an *X*-network and $D\subseteq A$ be a subset of arcs. There exists a unique $K\subseteq V$ such that

(i) $$D\subseteq K$$,

(ii) *K* is strongly closed, and

(iii) for every strongly closed $C\subseteq A$ such that $D\subseteq C$, it follows that $K\subseteq C$.

Thus *K* is the unique minimal strongly closed subset of *A* containing *D*.

### Proof

We define a sequence $D_0, D_1, \cdots , D_j, \cdots$ of subsets of *A*. Let $D_0=D$ and $n=0$. Let $\sim _n = \sim _{D_n}$.

If $D_n$ is not strongly closed, there are vertices $u_0, u_1, \cdots , u_{2m}$ in *V* such that $a \sim _n u_0$, $(u_0,u_1)\in A$, $u_1\sim _n u_2$, $(u_2,u_3)\in A$, $u_3\sim _n u_4$, $\cdots$, $(u_{2m-2},u_{2m-1})\in A$, $u_{2m-1}\sim _n u_{2m}=b$, and in addition $a\sim _n b$, but not every arc $(u_{2k},u_{2k+1})$ lies in $D_n$. Let $D_{n+1}$ be obtained from $D_n$ by including also all the arcs $(u_{2k},u_{2k+1})$ for $0\le k \le m-1$. If $D_{n+1}$ is strongly closed, we are done. Otherwise replace *n* by $n+1$ and repeat the argument.

By construction $D_n \subsetneq D_{n+1}$. Since *A* is a finite set, the chain $D_0\subsetneq D_1\subsetneq D_2 \cdots$ must terminate with some $D_n$, at which point $D_n$ is strongly closed. Let $K=D_n$. Then *K* contains $D=D_0$ and is strongly closed. Moreover, any strongly closed set *C* that contains $D_j$ for any $j<n$ must necessarily also contain $D_{j+1}$ by the strong closure property. Hence, *C* must contain *K*. $\square$

If *D* is a set of arcs in the *X*-network *N*, the *strong closure* $K = K(D)$ of *D* is the smallest set *K* of arcs that contains *D* and is strongly closed. By Theorem 3.7*K* is uniquely determined.

The next theorem is the main result of this section.

### Theorem 3.8

Let $N=(V,A,\rho ,\phi )$ be an *X*-network. Assume $D\subseteq A$ contains no *X*-arc. Let *K*(*D*) be the strong closure of *D*. Let $M_D(N) = Q_{K(D)}(N)$. Then

(1) $M_D(N)$ is an acyclic *X*-network.

(2) Each equivalence class [*v*] of $\sim _{K(D)}$ is convex.

(3) The projection $\psi :N\rightarrow M_D(N)$ is a leaf-preserving CSD map.

### Proof

Write $\sim$ for $\sim _{K(D)}$. *K*(*D*) exists by Theorem 3.7 and is root-preserving by Theorem 3.5. It contains no *X*-arc since otherwise *D* would contain an *X*-arc. Then (1) follows from Theorem 3.6. Note *K*(*D*) is closed by Theorem 3.5. Hence (2) follows from Theorem 3.4. Then (3) follows from Theorem 3.3. $\square$

Call $M_D(N)= Q_{K(D)}(N)$ the *merged* acyclic *X*-network for *D*. Note that in general, some arcs not in *D* need to be merged to produce an acyclic network. We nevertheless call *D* the *merging set* for $M_D(N)$.

The strong closure *K*(*D*) can be computed in practice using the method of the proof of Theorem 3.7. For hand calculation the following is often easier: Given *N* and *D*, since *K*(*D*) must be closed by Theorem 3.6, we adjoin to *D* all arcs in any directed path between two vertices *u* and *v* such that $u\sim _D v$. If necessary, repeat the process. Call the resulting set of arcs *C*. When *C* cannot be enlarged in this way, we compute $Q_C(N)$. Let $\psi :N\rightarrow Q_C(N)$ be the projection CSD map. If $Q_C(N)$ has a cycle, add to *C* any arcs (*u*, *v*) in *N* such that $(\psi (u),\psi (v))$ is an arc on a cycle of $Q_C(N)$.

For Fig. 2, with the indicated *D*, we find *D* is closed. We then find $Q_D(N)$, also shown, where [8] represents [6,7,8] and [9] represents [9,10,11]. Let $\psi :N\rightarrow Q_D(N)$ be the CSD projection map. In $Q_D(N)$ there is a cycle [8], [9], [8]. Since (11,6) in *N* satisfies $(\psi (11),\psi (6))=([9],[8])$ in $Q_D(N)$, we must adjoin (11,6) to *D*. Since (8,9) in *N* satisfies $(\psi (8),\psi (9))=([8],[9])$ in $Q_D(N)$, we must adjoin (8,9) to *D*. Hence $C=\{(6,7), (8,7), (9,10), (11,10), (11,6), (8,9)\}$. We see $Q_C(N)$ is acyclic, so $K(D)=C$.

**Fig. 3:**
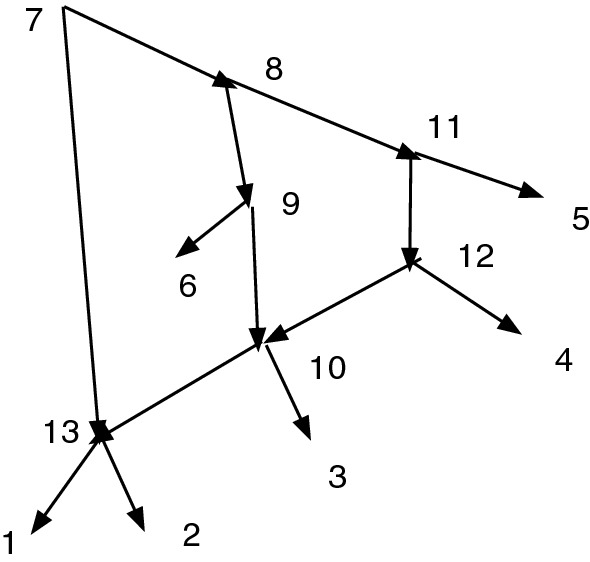
A network *N* containing two lengthening paths of the redundant arc (7,13)

For Fig. 3, suppose $D=\{(7,13)\}$. The path 7, 8, 9, 10, 13 shows that we must adjoin (7,8), (8,9), (9,10), (10,13), so now $C=\{(7,13), (7,8), (8,9), (9,10),$ $(10,13)\}$. But now 8 and 10 are vertices satisfying $8\sim _C 10$, so the path 8, 11, 12, 10 shows we must adjoin (8,11), (11,12), (12,10) to *C*. This enlarges *C* to $C'=\{(7,13), (7,8), (8,9), (9,10), (10,13), (8,11), (11,12), (12,10) \}$. This enlarged $C'$ is closed and $Q_{C'}(N)$ is acyclic, so $K(D)=C'$.

In the case where *N* is a cyclic *X*-network and $D=\emptyset$, we find $K(\emptyset )$ is nonempty using this procedure. On the other hand, if *N* is an acyclic *X*-network then $K(\emptyset )=\emptyset$.

### Theorem 3.9

Let $N=(V,A,\rho ,\phi )$ be an *X*-network. Assume $D\subseteq A$ contains no *X*-arc. Let $\psi :N\rightarrow M_D(N)$ be the projection. An arc $(a,b)\in A$ is in *K*(*D*) iff $\psi (a)=\psi (b)$.

### Proof

$M_D(N)=Q_{K(D)}(N)$ is defined utilizing the equivalence relation $\sim _{K(D)}$. If $(a,b)\in K(D)$ it follows that $a\sim _{K(D)} b$ so $[a]=[b]$, whence $\psi (a)=\psi (b)$. Conversely, suppose $(a,b)\in A$ and $\psi (a)=\psi (b)$. Since *K*(*D*) is strongly closed, it is closed by Theorem 3.6. By closure it follows that $(a,b)\in K(D)$. $\square$

## Deriving an SCD Network from *N*

This section gives a general construction, given an *X*-network *N*, to produce a uniquely determined acyclic *X*-network called $\text {SCD}(N)$ in which, for almost all arcs (*u*, *v*), the clusters are distinct (i.e., $cl(u)\ne cl(v)$). The only possible exceptions occur when *v* is a leaf. For complicated *N*, $\text {SCD}(N)$ can be very much simpler than *N*. Moreover, $d_{RF}(N,\text {SCD}(N))=0$.

We need to consider the behavior of clusters under contraction of arcs.

An acyclic *X*- network $N=(V,A,\rho ,\phi )$ is *successively cluster-distinct* (SCD) if, whenever $(u,v)\in A$, then $cl(v)\ne cl(u)$ unless for some $x\in X$, $v=\phi (x)$ and $u=p(x)$. The exception at the end is intended to make the definition consistent with the condition (N5), which often forces *p*(*x*) to have out-degree one and therefore $cl(p(x))=cl(\phi (x))=\{x\}$. (In Willson (2012) a network *N* was called SCD without the exception, but the networks there could fail (N5).)

In this section, we show that it is often easy to simplify a network *N* greatly so as to make it SCD.

Let $N=(V,A,\rho ,\phi )$ and let $\sim$ be a connected leaf-preserving and root-preserving equivalence relation on *V*. Suppose $a,b\in V$. A *generalized path* or *g-path* in *N* from *a* to *b* is a sequence of vertices $a, u_0, v_1, u_1, v_2, \cdots , u_{k-1}, v_k, b$ such that $a\sim u_0$, $(u_0,v_1)\in A$, $v_1\sim u_1$, $(u_1,v_2)\in A$, $v_2\sim u_2$, $(u_2,v_3)\in A$, $\cdots$, $v_{k-1}\sim u_{k-1}$, $(u_{k-1},v_k)\in A$, $v_k\sim b$.

In a g-path one always either utilizes an arc $(u_i,v_{i+1})\in A$, or else one stays within an equivalence class (but ignoring the direction of any arcs within the equivalence class).

### Lemma 4.1

Let $N=(V,A,\rho ,\phi )$ be an *X*-network, let $D\subseteq A$ contain no *X*-arc, and let $M_D(N)=(V',A',\rho ',\phi ')$. Let $\psi :N\rightarrow M_D(N)$ be the projection.

(1) Suppose there is a path in *N* from *v* to *w*. Then, there is a path in $M_D(N)$ from $\psi (v)$ to $\psi (w)$.

(2) $$cl(v;N)\subseteq cl(\psi (v);M_D(N))$$.

(3) If $u\sim _D v$ in *N*, then $cl(\psi (u))=cl(\psi (v))$.

(4) Suppose there is a path in $M_D(N)$ from [*a*] to [*b*]. Then in *N* there is a g-path from *a* to *b*.

### Proof

Write $\sim$ for $\sim _D$ and note $\psi (v)=[v]$.

(1). Let $v=u_0, u_1,\cdots , u_k=w$ be a path in *N*. Since $\psi$ is a CSD map the sequence $\psi (u)=\psi (u_0), \psi (u_1), \cdots , \psi (u_k)=\psi (w)$ of vertices satisfies that for each *i*, either $\psi (u_i)=\psi (u_{i+1})$ or else $(\psi (u_i), \psi (u_{i+1}))$ is an arc of $M_D(N)$. From this the result is clear.

(2) $cl(v;N)=\{x\in X: v \le \phi (x)$ in $M_D(N)\}$. By (1) if $x\in cl(v)$ it follows there is a path from $\psi (v)$ to $\psi (\phi (x)) = \phi '(x)$, so $x\in cl(\phi (v))$.

(3) Since $$u\sim v$$, $$\psi (u) = [u] = [v] = \psi (v)$$.

(4) Suppose $[a]=[w_0],[w_1],\cdots , [w_k]=[b]$ is a path in $M_D(N)$. For $i=0,\cdots , k-1$, since $([w_i],[w_{i+1}])\in A'$, we may choose $u_i\in [w_i]$, $v_{i+1}\in [w_{i+1}]$ such that $(u_i,v_{i+1})\in A$ because $\psi$ is a CSD map. Then $a, u_0, v_1, u_1, v_2, \cdots , u_{k-1}, v_k, b$ is a g-path because $[u_i]=[v_i]=[w_i]$ for $i=1,\cdots ,k-1$, and $[a]=[u_0]=[w_0]$, and $[b]=[w_k]=[v_k]$. $\square$

### Lemma 4.2

Let $N = (V,A, \rho , \phi )$ be an *X*-network. Let $D = \{(a,b) \in A: cl(a) = cl(b)$ and *b* is not a leaf$\}$. Then, *D* is strongly closed and contains no *X*-arcs.

### Proof

It is immediate that *D* contains no *X*-arcs since such were specifically excluded. Note that if $u\sim _D v$ then there exists a sequence $u=u_0, u_1, \cdots , u_k=v$ such that for each *i*, either $(u_i,u_{i+1})\in D$ or $(u_{i+1},u_i)\in D$. Since there is no *X*-arc in *D*, no $u_i$ is a leaf. Hence $cl(u)=cl(u_0)=cl(u_1)=\cdots = cl(u_k)=cl(v)$.

To see that *D* is strongly closed, suppose there are vertices $a, u_0, u_1, \cdots ,$ $u_{2m}=b$ in *V* such that $a \sim _D u_0$, $(u_0,u_1)\in A$, $u_1\sim _D u_2$, $(u_2,u_3)\in A$, $u_3\sim _D u_4$, $\cdots$, $(u_{2m-2},u_{2m-1})\in A$, $u_{2m-1}\sim _D u_{2m}=b$, and in addition $a\sim _D b$. We must show that each of the arcs $(u_{2k},u_{2k+1})$ lies in *D*. Then $cl(a)=cl(u_0)\supseteq cl(u_1)=cl(u_2)$ $\supseteq cl(u_3) = cl(u_4)$ $=\cdots \supseteq$ $cl(u_{2m-1}) =cl(u_{2m}) = cl(b)$. But since $a\sim _D b$, we know $cl(a)=cl(b)$ so the chain in inclusions must be a chain of equalities. Thus each $(u_{2k},u_{2k+1})\in D$. $\square$

### Theorem 4.3

Let $N = (V,A, \rho , \phi )$ be an *X*-network. Let $D = \{(a,b) \in A: cl(a) = cl(b)$ and *b* is not a leaf$\}$. Form $M_D(N)$, and let $\psi : N \rightarrow M_D(N)$ be the projection CSD map. Then

(1) $M_D(N)$ is an acyclic *X*-network.

(2) For every $$v \in V$$, $$cl(v; N) = cl(\psi (v); M_D(N))$$.

(3) For every arc $(a,b) \in A$, either $\psi (a) = \psi (b)$ or else the arc $(\psi (a), \psi (b))$ satisfies $cl(\psi (a)) \ne cl(\psi (b))$, or else *b* is a leaf.

(4) $M_D(N)$ is successively cluster-distinct (SCD).

(5) No vertex of $M_D(N)$ (other than possibly *p*(*x*) for some $x\in X$) has out-degree one.

(6) $$d_{RF}(N, M_D(N)) = 0$$.

### Proof

(1) follows from Theorem 3.8 and Lemma 4.2.

(2) By Lemma 4.1, $cl(v; N) \subseteq cl(\psi (v); M_D(N))$. Conversely, suppose $x \in cl(\psi (v); M_D(N))$. Let $M_D(N)=(V',A',\rho ',\phi ')$. There is a path in $M_D(N)$ from $\psi (v)$ to $\phi '(x)$. Since $\psi (\phi (x))=\phi '(x)$, by Lemma 4.1 there is a g-path in *N* from *v* to $\phi (x)$. Let $v, u_0, v_1, u_1, v_2, \cdots , u_{k-1}, v_k, \phi (x)$ be this g-path. Note $[v]=[u_0]$, $[\phi (x)]=[v_k]$, $[u_i]=[v_i]$ for $i=1,\cdots ,k-1$, and $(u_i,v_{i+1})\in A$ for $i=0,\cdots ,k-1$.

Since $\{\phi (x)\}=[\phi (x)]=[v_k]$, it follows $v_k=\phi (x)$. Since $(u_{k-1},v_k)\in A$, $x\in cl(u_{k-1})$. Since $v_{k-1}\sim u_{k-1}$, by the definition of *D* it follows that $cl(v_{k-1})=cl(u_{k-1})$, so $x\in cl(v_{k-1})$. Since $(u_{k-2},v_{k-1})\in A$, it follows $x\in cl(u_{k-2})$. Since $v_{k-2}\sim u_{k-2}$ it follows $cl(v_{k-2})=cl(u_{k-2})$, so $x\in cl(v_{k-2})$. We repeat the argument. By induction we find $x\in cl(u_0)$ and $cl(v)=cl(u_0)$ since $v\sim u_0$, so $x\in cl(v)$. This proves (2).

For (3) suppose $(a,b) \in A$ and *b* is not a leaf. If $\psi (a) \ne \psi (b)$, then $(\psi (a), \psi (b))$ is an arc of $M_D(N)$ since $\psi$ is a CSD map. Moreover, $cl(\psi (a)) = cl(a)$ and $cl(\psi (b)) = cl(b)$ by (2). Since $(a,b)\in A$, $cl(b) \subseteq cl(a)$. If $cl(b) = cl(a)$, then $(a,b) \in D$ so $\psi (a) = \psi (b)$, a contradiction. It follows that $cl(b) \ne cl(a)$, whence $cl(\psi (b)) \ne cl(\psi (a))$.

For (4) suppose (*u*, *v*) is an arc of $M_D(N)$ and *v* is not a leaf. There exists $(a,b)\in A$ such that $(u,v)=(\psi (a),\psi (b))$ since $\psi$ is a CSD map, and *b* is not a leaf since $\psi$ is leaf-preserving. Since $(\psi (a),\psi (b))$ is an arc, $cl(\psi (b))\subseteq cl(\psi (a))$, and $cl(\psi (a))\ne cl(\psi (b))$ by (3), proving that $M_D(N)$ is SCD.

For (5) write $M_D(N)=(V',A',\rho ',\phi ')$. Suppose a vertex $u\in A'$ has out-degree one with unique child *c*. Then $cl(u)=cl(c)$. Since $M_D(N)$ is SCD, it follows that for some $x\in X$, $c=\phi (x)$ and so $u=p(x)$ by (N5).

For (6), note $Cl(N)=Cl(M_D(N))$ by (2), using the fact that $\psi :V\rightarrow V'$ is surjective. $\square$

Recall that a vertex *v* is *trivial* if $indeg(v)=outdeg(v) = 1$. Write the SCD acyclic *X*-network $M_D(N)$ of Theorem 4.3 as $M_D(N)=(V',A',\rho ',\phi ')$. It is possible that $M_D(N)$ contains a trivial vertex *v* with unique child *c*. When this happens, $cl(c)=cl(v)$, and, by Theorem 4.3(5), for some $x\in X$, $c=\phi '(x)$ and $v=p(x;M_D(N))$. Such trivial vertices are a nuisance and it is easy to remove them. Since *p*(*x*) is trivial, it has a unique parent *u*(*x*). By Theorem 4.3, *u*(*x*) satisfies $outdeg(u(x))>1$ and $cl(u(x))\ne cl(p(x))$. Hence, the trivial vertex *p*(*x*) can be merged with *u*(*x*) and hence removed. We state this as a theorem:

### Theorem 4.4

Suppose $N=(V,A,\rho ,\phi )$ is an *X*-network and $M_D(N)=(V',A',\rho ', \phi ')$ is the acyclic SCD network of Theorem 4.3. Write *p*(*x*) for $p(x;M_D(N))$. Let

$$\begin{aligned} E=\{(u(x),p(x))\in A': x\in X, p(x)\text { is trivial and } u(x) \text{ is } \text{ its } \text{ unique } \text{ parent }\}. \end{aligned}$$

Define $$\text {SCD}(N)=M_E(M_D(N))$$. Then

(1) $\text {SCD}(N)$ is an acyclic SCD *X*-network.

(2) There is a leaf-preserving CSD map $\psi :N\rightarrow \text {SCD}(N)$.

(3) $\text {SCD}(N)$ contains no trivial vertices.

(4) $\text {SCD}(N)$ is a phylogenetic *X*-network.

(5) $$\text {SCD}(N)$$ satisfies $$d_{RF}(N,\text {SCD}(N))=0$$.

### Proof

It is immediate that *E* contains no *X*-arcs. It is easy to see that *E* is strongly closed. Hence, $\text {SCD}(N)$ is an acyclic *X*-network, proving part of (1).

There are leaf-preserving CSD maps $\psi _1:N\rightarrow M_D(N)$ and $\psi _2:M_D(N)\rightarrow \text {SCD}(N)$, so their composition $\psi = \psi _2 \circ \psi _1$ is a leaf-preserving CSD map from *N* to $\text {SCD}(N)$, proving (2). We see $p(x;\text {SCD}(N)) = [u(x)]$; since *u*(*x*) did not have out-degree one in $M_D(N)$, [*u*(*x*)] does not have out-degree one in $\text {SCD}(N)$ and [*u*(*x*)] is not trivial. Thus, $\text {SCD}(N)$ has no trivial vertices, proving (3). Note $cl([u(x)];\text {SCD}(N)) = cl(u(x);M_D(N))$, so $\text {SCD}(N)$ is SCD and $Cl(\text {SCD}(N))=Cl(M_D(N))=Cl(N)$, completing the proof of (1) and proving (5). Then (4) follows from (1) and (3). $\square$

A very similar network was described in Willson (2012) by a different approach.

### Example 1

**Fig. 4:**
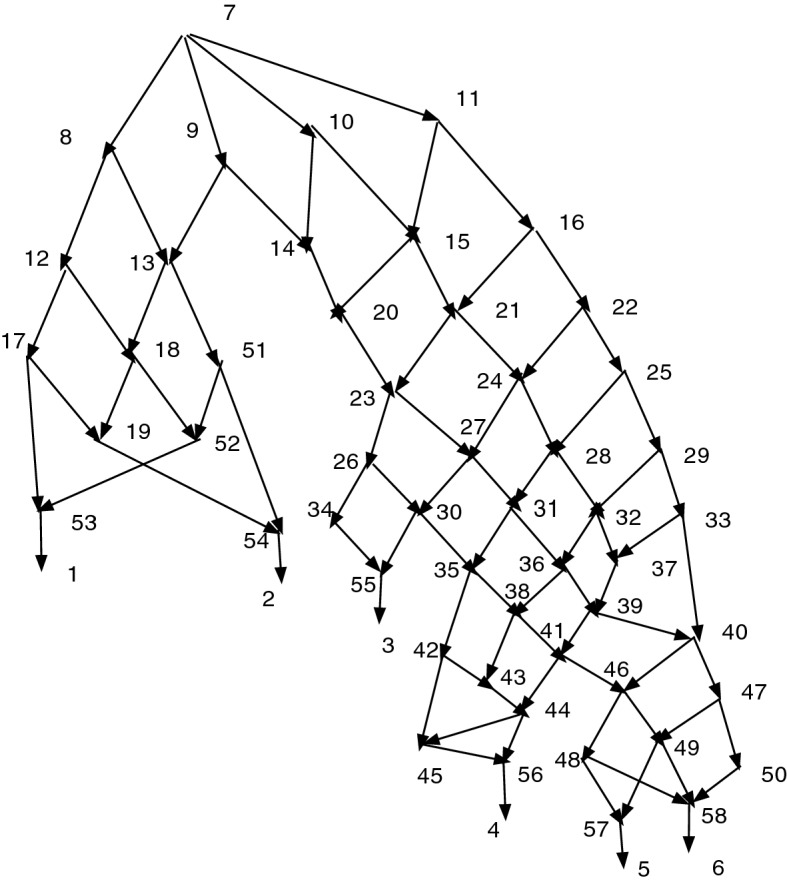
A network *N* that greatly simplifies to $\text {SCD}(N)$ (shown in Fig. 5) if $D = \{(a,b) \in A: cl(a) = cl(b)$ and *b* is not a leaf$\}$ and trivial vertices are removed

**Fig. 5:**
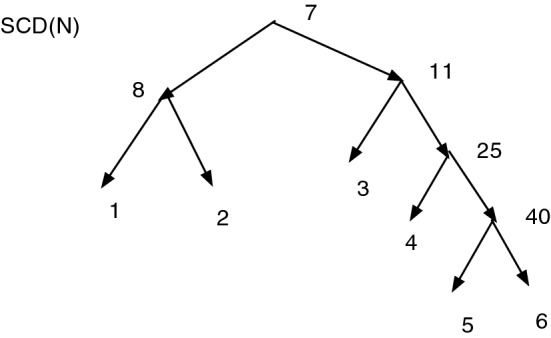
The network $\text {SCD}(N)$ for the network in Fig. 4

Figure 4 gives an example of an *X*-network *N*, and Fig. 5 shows $\text {SCD}(N)$. In this case $\text {SCD}(N)$ is a tree, clearly indicating the main features of *N* and much simpler than *N*. Vertices in $\text {SCD}(N)$ are labeled by a representative vertex of *N* with the same cluster.

## Removing Redundant Arcs from an *X*-Network

Our goal in this paper is the construction of normal networks which by definition contain no redundant arcs. A crucial step will be removing from an *X*-network *N* all its redundant arcs to form $\text {R}(N)$. This short section studies this process. Unfortunately, the natural map $\psi :N\rightarrow \text {R}(N)$ is not a CSD map unless $N=\text {R}(N)$, causing complications later in this paper.

If *N* is an *X*-network, let $\text {R}(N)$ denote the directed graph obtained from *N* by removing all redundant arcs. More precisely, if $N = (V,A,\rho ,\phi )$ then $\text {R}(N) = (V,A',\rho ,\phi )$ where $A'$ is obtained from *A* by removing all arcs redundant in *N*.

### Theorem 5.1

Suppose $N = (V,A,\rho , \phi )$ is an acyclic *X*-network. Then $\text {R}(N)$ is an acyclic *X*-network.

The proof is straight-forward and is omitted.

**Fig. 6:**
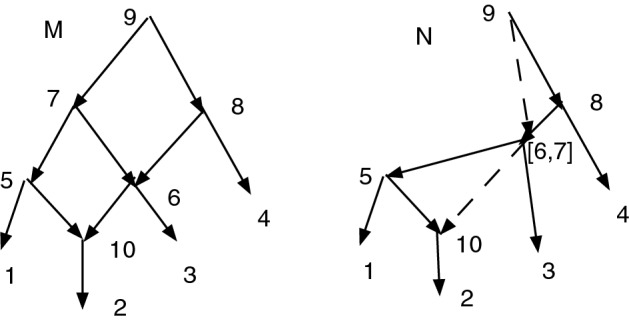
There is no CSD map from $\text {R}(M)$ to $\text {R}(N)$ nor from *N* to $\text {R}(N)$

If $f: M \rightarrow N$ is a CSD map, then *f* may not induce a CSD map from $\text {R}(M)$ to $\text {R}(N)$. Figure 6 shows an *X*-network *M*. Let $D=\{(7,6)\}$ and $N=Q_D(M)=M_D(M)$, also shown. *M* has no redundant arcs, while *N* has two redundant arcs, indicated by dashes. The projection map $\psi : M \rightarrow N$ is a CSD map. But $\text {R}(M) = M$, $\text {R}(N)$ is *N* without the dashed arcs, and $\psi : \text {R}(M) \rightarrow \text {R}(N)$ is not a CSD map since (6,10) is an arc in $\text {R}(M)$ but $\psi (6,10)=([6,7],10)$ and there is no such arc in $\text {R}(N)$. Indeed, it is easy to see that there is no CSD map $f: \text {R}(M) \rightarrow \text {R}(N)$.

In the same figure, one sees easily that there is no CSD map from *N* to $\text {R}(N)$.

### Theorem 5.2

Suppose *N* is an acyclic *X*-network. For each $v \in V$ $cl(v;\text {R}(N)) = cl(v;N)$. Moreover, $Cl(\text {R}(N)) = Cl(N)$ and $d_{RF}(N,\text {R}(N))=0$.

### Proof

Suppose $x \in cl(v;N)$. There is a path from *v* to $\phi (x)$ in *N*. By Theorem 2.1 a path from *v* to $\phi (x)$ in *N* of maximal length contains no redundant arc, hence lies in $\text {R}(N)$. It follows that $x \in cl(v;\text {R}(N))$. Conversely, suppose $x \in cl(v; \text {R}(N))$. There is a path in $\text {R}(N)$ from *v* to $\phi (x)$, so the same path is a path in *N* from *v* to *x*, proving $x \in cl(v;N)$. Hence $cl(v;\text {R}(N)) = cl(v;N)$. The rest follows easily. $\square$

## Generalized Wired Lifts

Let $N = (V,A,\rho ,\phi )$ and $N' = (V',A', \rho ', \phi ')$ be *X*-networks. Suppose $\psi : N \rightarrow N'$ is a CSD map. In Willson (2012) a *wired lift* of $N'$ into *N* is described. It provides a method for visualizing $N'$ within *N*. In this section we modify and generalize the notion so that $\psi$ does not quite need to be a CSD map but is only a *connected* map. This will let us obtain wired lifts from a process that includes both CSD maps and removing redundant arcs.

Let $N=(V,A,\rho ,\phi )$ and $N'=(V',A',\rho ',\phi ')$ be *X*-networks, and let $f:V\rightarrow V'$. We say *f* is a *connected* map $f:N\rightarrow N'$ if

(K1) $$f:V\rightarrow V'$$ is surjective,

(K2) $$f(\rho )=\rho '$$,

(K3) for all $$x\in X$$ $$f(\phi (x))=\phi '(x)$$,

(K4) for each $(u',v')\in A'$ there exists $(u,v)\in A$ such that $f(u)=u'$ and $f(v)=v'$,

(K5) for each $v'\in V'$ the set $f^{-1}(v')$ is connected.

It is immediate that a CSD map is connected, but a connected map need not be CSD. Suppose $f_1:N_1\rightarrow N_2$ and $f_2:\text {R}(N_2)\rightarrow N_3$ are CSD maps. Since $V(N_2)=V(\text {R}(N_2))$ the composition $f=f_2\circ f_1$ of $f_1:V(N_1)\rightarrow V(N_2)$ and $f_2:V(\text {R}(N_2))\rightarrow V(N_3)$ is defined. We shall see in Theorem 6.4 that *f* is a connected map while in general it is not a CSD map.

Let $f:N\rightarrow N'$ be a connected map, and let $2^V$ denote the set of subsets of *V*. A *(generalized) wired lift* of *f* (or of $N'$ into *N*) is a pair $(f^{-1},E_1)$ where $f^{-1}$ is the map $f^{-1}:V'\rightarrow 2^V$ given by $f^{-1}(v')$ and where $E_1\subseteq A$ satisfies the following two conditions:

(W1) For each arc $(u,v)\in E_1$, $f(u)\ne f(v)$ and $(f(u),f(v))\in A'$. Denote $f(u,v)=(f(u),f(v))$.

(W2) For every arc $(u',v')\in A'$, there exists $(u,v)\in E_1$ such that $f(u,v)=(u',v')$. We will say the arc (*u*, *v*) *represents* $(u',v')$ or is a *pre-arc* of $(u',v')$.

Call the members of $E_1$ the *representative arcs* since each represents an arc of $A'$.

Note that the collection of all $f^{-1}(v')$ for $v'\in V'$ is a partition of *V*. Thus for all $v'\in V'$, $f^{-1}(v')\ne \emptyset$; if $u'\ne v'$ are in $V'$, then $f^{-1}(u')\cap f^{-1}(v')=\emptyset$; and $\cup f^{-1}(v') = V$ where the union is over all $v'\in V'$.

Suppose $f:N\rightarrow N'$ is a CSD map. A *backwards map* *g* is a map $g:A' \rightarrow A$ which satisfies that, for all $(u',v')\in A'$, if $(u,v) = g(u',v')$ then $(f(u),f(v))= (u',v')$. Thus $f(g(u',v'))= (u',v')$ for all $(u',v')\in A'$. Since *f* is a CSD map, for each $(u',v')\in A$ such a (*u*, *v*) exists, and $g(u',v')$ provides a unique choice of a pre-arc of $(u',v')$.

There are several situations that give rise to wired lifts. We describe three of them in the next theorem. A fourth will be given in Theorem 6.4.

### Theorem 6.1

Let $N=(V,A,\rho ,\phi )$ and $N'=(V',A',\rho ',\phi ')$ be *X*-networks, and let $f:V\rightarrow V'$. Suppose $f:N\rightarrow N'$ is a CSD map.

(1) Let $E_1=\{(u,v)\in A: f(u)\ne f(v)\}$. Then $(f^{-1},E_1)$ is a wired lift of $N'$.

(2) Suppose $g:A' \rightarrow A$ is a backwards map. Let $E_1 = \{g(u',v'): (u',v')\in A'\}$. Then $(f^{-1},E_1)$ is a wired lift of $N'$.

(3) Let $\text {R}(N')=(V',A'',\rho ',\phi ')$ be the result of removing all redundant arcs from $N'$. Let $E_1=\{(u,v)\in A: f(u)\ne f(v) \text { and } (f(u),f(v))\in A''\}$. Then $(f^{-1},E_1)$ is a wired lift of $\text {R}(N')$.

### Proof

(1) and (2) are immediate from the definitions since a CSD map is connected. For (3), note that $V(N')=V(\text {R}(N'))$, so the map *f* can be regarded as a map $f:N\rightarrow \text {R}(N')$. This map will not be a CSD map if $N'$ has any redundant arcs, but it is a connected map. Then (3) follows. $\square$

**Fig. 7:**
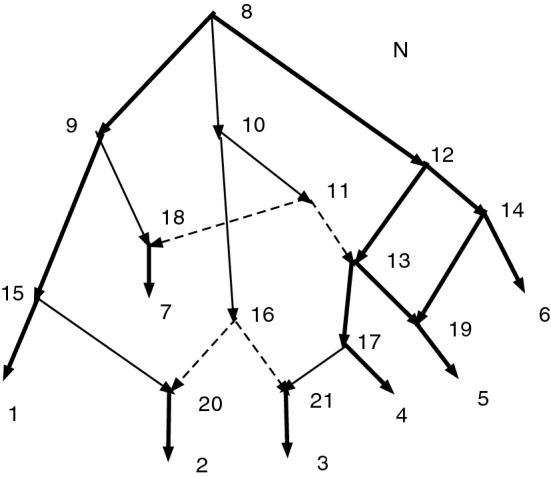
An example of a wired lift. Thin solid arcs indicate identification of the vertices and can be followed in either direction. Wide solid arcs must be followed in their direction. Dashed arcs cannot be used in g-paths

Given a connected map $f:N\rightarrow N'$, a wired lift $(f^{-1},E_1)$ can be visualized using a diagram of *N*. An example is shown below in Fig. 7. The diagram is exactly the diagram of *N* except that each arc may be wide solid, thin solid, or thin dashed. Suppose $N=(V,A,\rho ,\phi )$. For every arc $(u,v)\in A$ such that $f(u)\ne f(v)$ draw (*u*, *v*) a wide solid arrow if $(u,v)\in E_1$ and a thin dashed arrow if $(u,v)\notin E_1$. For each arc $(u,v)\in A$ such that $f(u)=f(v)$ draw the arc as a thin solid arrow. (If color is available, one might instead color red the arcs $(u,v)\in A$ satisfying $f(u)=f(v)$ for vividness.) Thin solid arcs make the sets $f^{-1}(v')$ apparent in *N* and each vertex of $N'$ corresponds to a connected component of the thin solid arcs. Each arc $(u',v')\in A'$ has a corresponding wide solid arc $(u,v)\in A$, justifying the word “lift”. The “wires” are the thin solid arcs. Paths in $N'$ can be recognized in the wired lift as *g-paths* using *allowed steps*, which we will now describe.

Let $N=(V,A,\rho ,\phi )$ and $N'=(V',A',\rho ',\phi ')$ be *X*-networks, with $f:V\rightarrow V'$ a connected map, and suppose $(f^{-1},E_1)$ is a wired lift of *f*. If *u* and *v* are in *V*, we say there is an *allowed step* from *u* to *v* if either $(u,v)\in E_1$, or ($(u,v)\in A$ and $f(u)=f(v)$), or ($(v,u)\in A$ and $f(u)=f(v)$). Note that the step either follows a wide solid arc in $E_1$ forwards or else follows a thin solid arc, possibly forwards, possibly backwards. Dashed arcs cannot be used.

### Theorem 6.2

Let $N=(V,A,\rho ,\phi )$ and $N'=(V',A',\rho ',\phi ')$ be *X*-networks, with $f:V\rightarrow V'$ a connected map, and suppose $(f^{-1},E_1)$ is a wired lift of *f*. Let $\sim$ be the equivalence relation on *V* defined by $u\sim v$ if and only if $f(u)=f(v)$. Suppose $a,b\in V$. The following are equivalent:

(1) In *N* there is a sequence of vertices $a, u_0, v_1, u_1, v_2, \cdots , u_{k-1}, v_k, b$ such that $a\sim u_0$, $(u_0,v_1)\in E_1$, $v_1\sim u_1$, $(u_1,v_2)\in E_1$, $v_2\sim u_2$, $(u_2,v_2)\in E_1$, $\cdots$, $v_{k-1}\sim u_{k-1}$, $(u_{k-1},v_k)\in E_1$, $v_k\sim b$.

(2) There is a sequence of vertices $a= u_0, u_1, u_2, \cdots , u_{k-1}, u_k= b$ in *N* such that, for *i* such that $0\le i\le k-1$, there is an allowed step from $u_i$ to $u_{i+1}$.

### Proof

Suppose there is a sequence of type (1). If $u\sim v$ then since $f(u)=f(v)$ and $f^{-1}(f(u))$ is connected, there is a sequence $u=w_0, w_1, \cdots , w_m=v$ such that each $w_i$ lies in $f^{-1}(f(u))$ and, for $0\le i\le m-1$, either $(w_i,w_{i+1})\in A$ or $(w_{i+1},w_i)\in A$. Thus, there is an allowed step from $w_i$ to $w_{i+1}$. Hence given a sequence of type (1), there is a sequence of type (2).

Conversely, given a sequence of type (2), if the allowed step from $u_i$ to $u_{i+1}$ satisfies $f(u_i)\ne f(u_{i+1})$ then $(u_i, u_{i+1})\in E_1$. If $f(u_j)=f(u_{j+1}) = \cdots = f(u_n)$ but $f(u_{j-1})\ne f(u_j)$ and $f(u_n)\ne f(u_{n+1})$ then we may replace $u_j, \cdots , u_n$ by simply $u_j\sim u_n$. Thus, there is a sequence of type (1). $\square$

We will call a sequence of either type a *generalized path* or *g-path* from *a* to *b* in $(f^{-1},E_1)$. For specification they may be called type (1) or type (2).

### Theorem 6.3

Let $N=(V,A,\rho ,\phi )$ and $N'=(V',A',\rho ',\phi ')$ be *X*-networks. Let $f:N\rightarrow N'$ be a connected map, and let $(f^{-1},E_1)$ be a wired lift of *f*.

(1) Suppose $a= u_0, u_1, u_2, \cdots , u_{k-1}, u_k= b$ is a g-path in *N* (of type (2)). Then $f(a)= f(u_0), f(u_1)$, $f(u_2), \cdots$, $f(u_{k-1}), f(u_k)= f(b)$ yields a path in $N'$, possibly by suppressing multiple successive copies of the same vertex.

(2) Suppose $a'=w_0', w_1', \cdots , w_k'=b'$ is a path in $N'$, $f(a)=a'$, and $f(b)=b'$. Then, there is a g-path in *N* from *a* to *b*.

### Proof

If there is an allowed step from $u_i$ to $u_{i+1}$, either $(u_i,u_{i+1})\in E_1$, in which case $(f(u_i),f(u_{i+1}))$ is an arc of $N'$ by (W1), or else $f(u_i)=f(u_{i+1})$, proving (1).

Conversely suppose $a'=w_0', w_1', \cdots , w_k'=b'$ is a path in $N'$, $f(a)=a'$, and $f(b)=b'$. For $0\le i \le k-1$, since $(w_i', w_{i+1}')\in A'$, by (W2) there exists $(y_i,z_{i+1})\in E_1$ such that $f(y_i)=w_i'$ and $f(z_{i+1})=w_{i+1}'$. Note $f(a) = f(y_0)$, $f(b)=f(z_k)$, and $f(z_i)=f(y_i)$. Hence $a, y_0, z_1, y_1, z_2, \cdots , y_{k-1}, z_k, b$ is a g-path (of type (1)), proving (2). $\square$

In Willson (2012) there was a backwards map and, instead of all arcs in $\psi ^{-1}(v')$, only the arcs in some spanning tree in $\psi ^{-1}(v')$ containing each vertex in $\psi ^{-1}(v')$ which lies on an arc in $E_1$ were included. But this feature is not essential.

### Example 2

Figure 7 shows a wired lift that arises from a connected map $f:N\rightarrow N'$. All the arcs and vertices are from *N*; thus if we ignore thickness and dashing and include all arcs with their indicated directions, whether thin, wide, or dashed, the diagram exhibits *N*. A vertex of $N'$ with more than one preimage may be identified with a connected component of thin solid arcs. It is also convenient to identify each vertex $v'\in V'$ by the members of $f^{-1}(v')$ inside square brackets. One sees immediately that the vertex *f*(10) of $N'$ satisfies $f^{-1}(f(10))=\{8,10,11,16\}$ (from the component of thin arcs). We shall designate it [8, 10, 11, 16] or less formally [10], the equivalence class of 10. Similarly *f*(15) has inverse image $f^{-1}(f(15))=\{15,20\}$ and is written [15, 20]. Other vertices include [9, 18] and [17, 21]. Still other vertices have singleton inverse images such as [13] with $f^{-1}(f(13))=\{13\}$, but the brackets may be omitted.

The dashed arcs are not permitted on g-paths, and wide solid arcs must be followed in their direction. Thin solid arcs can be followed in either direction. Thus, 16, 10, 8, 9, 15, 1 is a g-path showing that $N'$ has a path from *f*(16) to *f*(1). The corresponding path in $N'$ is formally written [8, 10, 11, 16], [9, 18], [15], [1] or informally as [16],[9],15,1. There is clearly no path in *N* from 16 to 1. Similarly the g-path 21,17,4 shows that in $N'$ there is a path from [21] to [4]. Thus $4\in cl(f(21);N')$.

Suppose $N_1$, $N_2$, and $N_3$ are *X*-networks. Suppose $f_1: N_1\rightarrow N_2$ and $f_2: \text {R}(N_2)\rightarrow N_3$ are CSD maps, where $f_2$ denotes a simplification of $\text {R}(N_2)$. Let $f:V(N_1)\rightarrow V(N_3)$ be the composition $f(v_1)=f_2(f_1(v_1))$. In general $f:N_1\rightarrow N_3$ is not a CSD map since there is no CSD map from $N_2$ to $\text {R}(N_2)$. The following result shows that *f* is nevertheless a connected map and there is a wired lift of *f*. Consequently, we are able to visualize simplifications of $\text {R}(N_2)$.

### Theorem 6.4

Suppose for $i=1,2,3$, we have $N_i=(V_i,A_i,\rho _i,\phi _i)$ is an *X*-network. Write $\text {R}(N_2)=(V_2,A'_2,\rho _2,\phi _2)$ where $A'_2$ is the set of arcs in $A_2$ which are not redundant in $N_2$. Suppose $f_1: N_1\rightarrow N_2$ and $f_2: \text {R}(N_2)\rightarrow N_3$ are CSD maps. Let $f:V_1\rightarrow V_3$ be the composition of the vertex maps, $f(v_1)=f_2(f_1(v_1))$. Thus, $f^{-1}(v_3)= \{v_1\in V_1: f(v_1)=v_3\}$ for $v_3\in V_3$. Define $E_1=\{(u_1,v_1)\in A_1$:

(i) $$f_1(u_1)\ne f_1(v_1)$$ and $$(f_1(u_1), f_1(v_1))\in A'_2$$ and

(ii) $f(u_1) \ne f(v_1)$ and $(f(u_1), f(v_1))\in A_3\}$. Then

(1) $f:N_1\rightarrow N_3$ is a connected map.

(2) $(f^{-1}, E_1)$ is a wired lift of *f*.

### Proof

To see (1), note that *f* is well defined since $N_2$ and $\text {R}(N_2)$ have the same vertex set $V_2$. (K1), (K2), and (K3) are immediate. To see (K4) assume $(u_3,v_3)\in A_3$. Since $f_2$ is CSD, there exists $(u_2,v_2)\in A_2'$ such that $f_2(u_2)=u_3$ and $f_2(v_2)=v_3$. But $A_2'\subseteq A_2$ and $f_1$ is CSD. Hence there exists $(u_1,v_1)\in A_1$ such that $f_1(u_1)=u_2$ and $f_1(v_1)=v_2$. Thus $f(u_1)=u_3$ and $f(v_1)=v_3$, proving (K4). The argument for (K5) is the same as that of Theorem 3.3 in Willson (2012), used to prove that the composition of CSD maps is CSD. This completes the proof of (1).

For (2), to prove (W1) suppose $(u_1,v_1)\in E_1$. By (i) $(f_1(u_1), f_1(v_1))\in A'_2$, so since $f_2$ is a CSD map either $f(u_1)=f(v_1)$ or $(f(u_1),f(v_1))\in A_3$. The latter applies by (ii), proving (W1).

For (W2), given any arc $(u_3,v_3)\in A_3$ there exists $(u_2,v_2)\in A'_2$ such that $(f_2(u_2),f_2(v_2))=(u_3,v_3)$ since $f_2$ is a CSD map. Then since $f_1$ is a CSD map, there exists $(u_1,v_1)\in A_1$ such that $(f_1(u_1),f_1(v_1)) = (u_2,v_2)$. Hence for every $(u_3,v_3)\in A_3$ there exists $(u_1,v_1)\in E_1$ such that $(f(u_1),f(v_1))=(u_3,v_3)$. This proves (W2) and hence (2). $\square$

In the situation of Theorem 6.4, to draw the wired lift of *f* on the diagram of $N_1$, it follows that, for every arc $(u,v)\in A_1$, we draw the arc in one of three ways:

(1) a thin solid arc (*u*, *v*) if $f(u)=f(v)$,

(2) a thin dashed arc (*u*, *v*) if $f(u)\ne f(v)$ and $(f_1(u),f_1(v))$ is a redundant arc in $N_2$,

(3) a wide solid arc (*u*, *v*) if $f(u)\ne f(v)$ and $(f_1(u),f_1(v))$ is an arc in $N_2$ that is not redundant.

## Deriving a Normal Network from an *X*-Network

This section concerns methods, given an *X*-network *N*, to produce an acyclic *X*-network $M_D(N)$ with desirable properties. Often, an important step may be to remove redundant arcs, thus obtaining $\text {R}(M_D(N))$.

In particular, we shall want to find *D* such that $\text {R}(M_D(N))$ is normal. Call a network *N* *pre-normal* if $\text {R}(N)$ is normal. Thus, we seek *D* such that $M_D(N)$ is pre-normal.

Let *N* be an *X*-network. A vertex *v* of *N* is a *pre-normal obstacle* or (more simply) an *obstacle* if (1) *v* is not a leaf, and (2) every child of the vertex *v* in $\text {R}(N)$ is hybrid. Thus *v* may be regarded as an “obstacle” to $\text {R}(N)$ being tree-child. Since $\text {R}(N)$ contains no redundant arcs, this is an “obstacle" to $\text {R}(N)$ being normal, or equivalently to *N* being pre-normal.

We must ignore redundant arcs when deciding our strategies concerning which arcs to merge. To make these decisions we need to have a notion of in-degree and out-degree that does not count redundant arcs.

Suppose *v* is a vertex of an *X*-network *N*. The *non-redundant in-degree* of *v*, denoted *nrindeg*(*v*), is the number of non-redundant arcs (*p*, *v*); hence it is the number of parents of *v* by non-redundant arcs. If $v\ne \rho$ then by Theorem 2.1$nrindeg(v) \ge 1$. Clearly $nrindeg(v) \le indeg(v)$. The *non-redundant out-degree* of *v*, denoted *nroutdeg*(*v*), is the number of non-redundant arcs (*v*, *c*), hence the number of children of *v* by non-redundant arcs. If *v* is not a leaf, then by Theorem 2.1 it has a non-redundant child, whence $nroutdeg(v)\ge 1$.

A vertex *v* of *N* is *nonr-hybrid* if $nrindeg(v;N) \ge 2$. A vertex *c* is a *nonr-child* of *v* if (*v*, *c*) is a non-redundant arc; we also say *v* is a *nonr-parent* of *c*. A nonr-child *c* of *v* is a *nonr-tree-child* of *v* if $nrindeg(c) = 1$. A path $u_0, u_1, \cdots , u_k$ is a *nonr-path* if no arc $(u_i,u_{i+1})$ is redundant, for $0\le i\le k-1$.

It is immediate that *v* is a pre-normal obstacle iff (1) *v* is not a leaf, and (2) every nonr-child of *v* is nonr-hybrid.

**Fig. 8:**
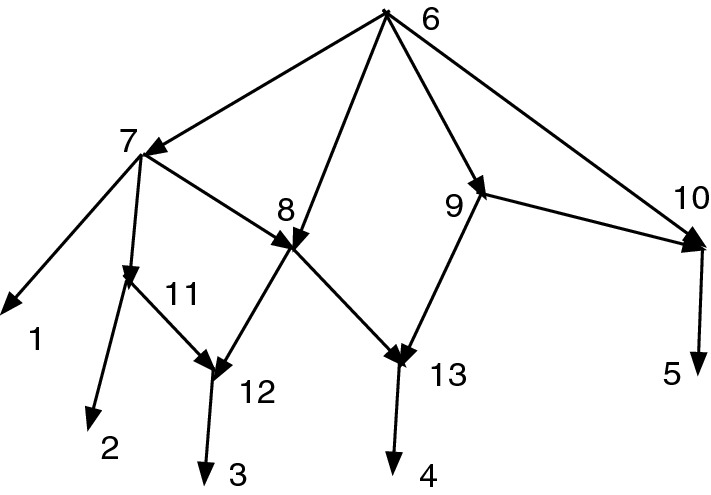
An *X*-network *N* with redundant arcs (6,8) and (6,10). Vertex 8 is an obstacle but 9 is not

Figure 8 shows an acyclic *X*-network with redundant arcs. Note that 8 is an obstacle since both its children 12 and 13 are nonr-hybrids. But 9 is not an obstacle since the only nonr-parent of 10 is 9 and $nrindeg(10)=1$.

An *X*-network *N* is *obstacle-free* if it contains no pre-normal obstacle.

### Theorem 7.1

Suppose *N* is an acyclic *X*-network that is obstacle-free. Then $\text {R}(N)$ is a normal *X*-network and *N* is pre-normal.

### Proof

By hypothesis, for every vertex *v* that is not a leaf, there is a non-redundant arc (*v*, *c*) with $nrindeg(c) = 1$. It follows that in $\text {R}(N)$, *c* is a tree-child of *v*. Since $\text {R}(N)$ has no redundant arcs, it follows that $\text {R}(N)$ is normal and *N* is pre-normal. $\square$

Theorem 7.1 further justifies the use of the term “pre-normal obstacle”. It is easy to see that a tree-child *X*-network is always pre-normal, but a pre-normal network need not be tree-child.

Theorem 7.1 suggests our strategy for normalization: Given an arbitrary *X*-network *N*, when we seek a normal network *M*, we know by Lemma 2.2 that *S*(*M*) will be regular; by (1) in the definition of regular network, *S*(*M*) is SCD. We are therefore seeking a network that is very close to being an SCD network, and it is plausible to start with the very general SCD network $\text {SCD}(N)$. We then recursively remove obstacles until there are no obstacles remaining. Next we remove redundant arcs to obtain a normal network. If we seek to obtain a uniquely determined normalization we are careful not to make arbitrary choices about which arcs to merge.

Now we show that there are different types of pre-normal obstacles. Let *N* be an acyclic *X*-network. Suppose *c* is an obstacle. An *allowable 1-fold parent chain* of *c* is a path $p^1,c$ such that $(p^1,c)$ is not redundant and $p^1$ has a nonr-tree-child $d \ne c$ (so $nrindeg(d)=1$, whence necessarily every other parent of *d* is via a redundant arc). An obstacle *c* is of *type 1* if *c* has an allowable 1-fold parent chain. If *c* has type 1 and $p^1,c$ is an allowable 1-fold parent chain, let $Dc(p^1,c) = \{(p^1,c)\}$.

Suppose *c* is an obstacle and $k>1$ is an integer. An *allowable k-fold parent chain* for *c* is a nonr-path $p^k, p^{k-1}, \cdots , p^1, p^0=c$ such that $p^k$ has a nonr-tree-child *d* distinct from $p^{k-1}$. An obstacle *c* is of *type k* if

(a) *c* is not of type $1, \cdots , k-1$; and

(b) *c* has an allowable *k*-fold parent chain.

In this situation, for this *k*-fold parent chain write

$$Dc(p^k,p^{k-1}, \cdots , c)$$ $$= \{(p^k,p^{k-1}), (p^{k-1}, p^{k-2}), \cdots , (p^1, c)\}$$.

### Theorem 7.2

Let *N* be an acyclic *X*-network. Then every pre-normal obstacle *c* has a unique type.

### Proof

It is clear that the type, if it exists, is unique.

Consider a path from $\rho$ to *c* which has maximal length *k*. Write this path as $u_0=\rho , u_1,$ $\cdots , u_k = c$. By Theorem 2.1 this is a nonr-path. If $\rho$ has a nonr-child *d* other than $u_1$, then this path is an allowable *k*-fold parent chain of *c*, so *c* has type at most *k*. If, instead, $u_1$ is the only nonr-child of $\rho$, then every other child *q* of $\rho$ satisfies that $(\rho ,q)$ is redundant. There is a lengthening nonr-path $\rho =v_0,v_1,\cdots , v_m=q$ by Theorem 2.1, whence $v_1$ is a nonr-child of $\rho$; since $u_1$ is the only such nonr-child, it follows $v_1 = u_1$. Indeed, every nonr-path from $\rho$ to any vertex other than $\rho$ or $u_1$ must begin with $\rho , u_1$. If $u_1$ has a nonr-child *d* other $u_2$, then $u_1, u_2, \cdots , c$ is an allowable $(k-1)$-fold parent chain and *c* has type $\le k-1$. Otherwise $u_2$ is the only nonr-child of $u_1$. Thus any nonr-path from $\rho$ to a vertex other than $\rho , u_1, u_2$ must begin $\rho , u_1, u_2$. We repeat the argument. If at any stage we have r such that $u_r$ has a nonr-child $d \ne u_{r+1}$, then $u_r, u_{r+1}, \cdots , c$ is an allowable $(k-r)$-fold parent chain. Otherwise every nonr-path from $\rho$ to a vertex other than $\rho , u_1, \cdots , u_r$ must start with $\rho , u_1, \cdots , u_r$.

If no such $r<k$ occurs, then we find that $\rho , u_1, \cdots , u_k=c$ is a nonr-path and every nonr-path from $\rho$ to any vertex other than $\rho , u_1, \cdots , c$ must begin with $\rho , u_1, \cdots , c$. But *c* is not a leaf hence must have a nonr-child *e*. Since *c* is an obstacle, $nrindeg(e) \ge 2$ so *e* has a nonr-parent $q\ne c$. Every nonr-path from $\rho$ to *q* must start $\rho , u_1, \cdots , c$, so there is a nonr-path from *c* to *q*, hence a nonr-path from *c* to *q* to *e*, showing that (*c*, *e*) is redundant, a contradiction. Hence some such $r<k$ must occur, and *c* has an allowable $(k-r)$-fold parent chain. $\square$

The following result shows a simple way to remove a type 1 obstacle:

### Lemma 7.3

Suppose *N* is an acyclic *X*-network and *c* is a type 1 obstacle with allowable 1-fold parent chain *p*, *c*, where *p* has nonr-tree-child $d\ne c$. Let $D = \{(p,c)\}$. Form $M_D(N)$ and let $\psi :N\rightarrow M_D(N)$ be the projection. Then $\psi (c)$ is not an obstacle in $M_D(N)$.

### Proof

Since *c* is an obstacle, it is not a leaf, so *D* contains no *X*-arc. Moreover, *D* is strongly closed since (*p*, *c*) is not redundant, and $Q_D(N)=M_D(N)$ is an acyclic *X*-network. Note $\psi (c)=[p,c]$ and in $M_D(N)$ there is an arc ([*p*, *c*], *d*). If *q* is any parent of *d* in *N* other than *p*, then (*q*, *d*) is redundant since *d* is a nonr-tree-child of *p*. Hence by Theorem 2.1 it has a lengthening of maximal length, ending with a non-redundant arc into *d*. Thus the lengthening must include the nonr-parent *p* of *d* and there is a path in *N* with non-redundant arcs from *q* to *p* to *d*. Since $\psi$ is a CSD map, there is a path in $M_D(N)$ from $q=\psi (q)$ to $\psi (p)=\psi (c)$ to $\psi (d)=d$, showing that (*q*, *d*) is redundant in $M_D(N)$, so $nrindeg(d; M_D(N))=1$. $\square$

The result above often generalizes to obstacles of type *k*. The next result assumes for simplicity that *D* is strongly closed.

### Lemma 7.4

Let *N* be an acyclic *X*-network with pre-normal obstacle *c* of type *k*. Suppose $p^k, ...., c$ is an allowable *k*-fold parent chain, where $p^k$ has nonr-tree-child $d \ne p^{k-1}$. Let $D = Dc(p^k, \cdots , c)$ $= \{ (p^k,p^{k-1}), (p^{k-1}, p^{k-2}), \cdots , (p^1, c)\}$. Assume *D* is strongly closed. Form $M_D(N)$ and let $\psi :N \rightarrow M_D(N)$ be the projection. Then $\psi (c)= [p^k, p^{k-1}, \cdots , c]$ has nonr-child *d* and $nrindeg(d; M_D(N)) = 1$, so $\psi (c)$ is not an obstacle in $M_D(N)$.

### Proof

Note that *D* contains no *X*-arcs since *c* is not a leaf. By Lemma 7.3, when we identify $p^k$ and $p^{k-1}$, *d* becomes a nonr-tree-child of $[p^k,p^{k-1}]$. When we next identify $[p^k,p^{k-1}]$ with $p^{k-2}$, *d* becomes a nonr-tree-child of $[p^k,p^{k-1}, p^{k-2}]$. This continues until we conclude that $\psi (c)$ has the nonr-tree-child *d*. $\square$

The following lemma shows that, often, once an obstacle is removed, it does not reappear when subsequent arcs are merged.

### Lemma 7.5

Suppose (*p*, *c*) is a non-redundant arc in the acyclic *X*-network *N* and $N'$ is obtained by identifying *p* and *c*. Let $\psi : N \rightarrow N'$ be the projection. Suppose (*a*, *b*) is a non-redundant arc in *N* and *b* is a nonr-tree-child of *a* (so $nrindeg(b;N) = 1$). Assume $b \ne p$, $b\ne c$. Then $(\psi (a),\psi (b))$ is a non-redundant arc of $N'$ and $\psi (b)$ has nonr-indegree 1.

### Proof

$(\psi (a),\psi (b))$ must be an arc unless $\psi (a) = \psi (b)$. But in $N'$ the only identification is $\psi (p)=\psi (c) = [p,c]$. If $\psi (a)=\psi (b)=[p,c]$, this would contradict that $b \ne p$, $b\ne c$, so $(\psi (a),\psi (b))$ is an arc in $N'$. If $(d,\psi (b))$ is a non-redundant arc in $N'$, then $d = \psi (a)$ since *a* is the only nonr-parent of *b* in *N*. Hence $nrindeg(\psi (b);N') = 1$. $\square$

It will be useful to remove trivial vertices which may have been created in the construction process. Suppose $N=(V,A,\rho ,\phi )$ is an *X*-network. Let $E = \{(u,v)\in A: v \text { is a trivial vertex} \}$. Define $\text {T}(N)=M_E(N)$. Note that *v* will have unique parent *u* and also a unique child since *v* is trivial. The next result shows that $\text {T}(N)$ has desirable properties.

### Theorem 7.6

Let $N=(V,A,\rho ,\phi )$ be an acyclic *X*-network. Then $\text {T}(N)$ is an acyclic *X*-network. Moreover

(1) $\text {T}(N)$ contains no trivial vertices and hence is a phylogenetic *X*-network.

(2) There is a leaf-preserving CSD map $\psi :N\rightarrow \text {T}(N)$.

(3) $$Cl(T(N))=Cl(N)$$.

(4) If *N* is normal, then $\text {T}(N)$ is normal.

### Proof

Note that *E* contains no *X*-arc because a leaf does not have out-degree one. Since *E* is clearly strongly closed, $\text {T}(N)$ is an acyclic *X*-network. (1) and (2) follow as in Theorem 4.4. (3) is obvious since if $(u,v)\in A$ and *u* is trivial, then $cl(u)=cl(v)$.

For (4) we first show that since *N* is tree-child, $N'=\text {T}(N)$ must also be tree-child. It suffices to prove this in the case where $N'$ is obtained from *N* by removing one trivial vertex *t* with parent *q* and child *c*. In $N'$ every non-leaf vertex except *q* obviously still has a tree-child, the same one as in *N*. We must show that *q* has a tree-child in $N'$. But *t* has a tree-child in *N* which must be *c* so *c* has no other nonr-parent than *t* in *N*. Hence *q* in $N'$ has child *c* which has no other nonr-parent and is therefore a tree-child. This proves $\text {T}(N)$ is tree-child.

For (4) we must also prove that $\text {T}(N)$ has no redundant arc. Again we may assume that $N'$ is obtained from *N* by the removal of a single trivial vertex *t* with parent *q* and child *c*. The only possible redundant arc in $N'$ is the new arc (*q*, *c*). If it is redundant, there is a path in $N'$ from *q* to *c* other than the arc, hence a path in *N* from *q* to *c* not through *t*. Such a path of maximal length by the proof of Theorem 2.1 contains no redundant arc, so *c* has a nonr-parent besides *t*. This contradicts that *N* was normal, since *t* in *N* has no tree-child. $\square$

We now show how, given an *X*-network *N*, to compute a uniquely determined normal *X*-network. We first compute a uniquely determined pre-normal acyclic *X*-network $\text {Prenorm}(N)$, which we call the *pre-normalization* of *N*. The computation uses the procedure PRENORM described below. Briefly, if *N* is not already a pre-normal acyclic *X*-network, we compute $N_1=\text {SCD}(N)$. If $N_1$ contains no obstacles then $\text {Prenorm}(N) = N_1$. Otherwise, for each obstacle *c* we compute its type *k* and find all the allowable *k*-fold parent chains for *c*. Let *D*(*c*) be the union of $Dc(p^k, \cdots , c) = \{(p^k, p^{k-1}), \cdots , (p^1, c)$ for all such allowable chains $p^k, \cdots , c$ for *c*. Let *D* be the union of the *D*(*c*) for all the obstacles *c*. We then compute $N_2 = M_D(N_1)$. If this has no obstacles then $\text {Prenorm}(N)=N_2$. If not, we repeat the process.

Here is a more detailed description of the computation of $\text {Prenorm}(N)$:
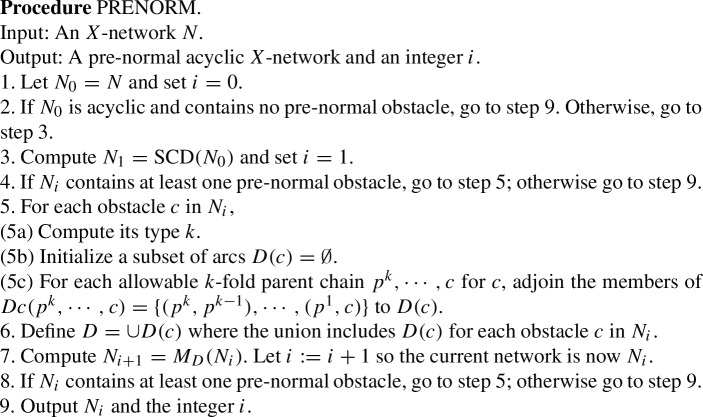


The network output by the procedure will be denoted $\text {Prenorm}(N)$. The integer output will be called the *height* of $\text {Prenorm}(N)$ and denoted *r*. Thus $N_r=\text {Prenorm}(N)$. Note that $r= 0$ if *N* is acyclic and pre-normal; otherwise, *r* is 1 more than the number of times the procedure passes through steps 5 , 6, 7. The height *r* is a crude indicator of the complexity of the calculation. The examples in this paper satisfy $r\le 2$, including those examples with real data. The author has worked examples with $r=3$.

The next theorem shows that the procedure works.

### Theorem 7.7

Let $N=(V,A,\rho ,\phi )$ be an *X*-network.

Apply procedure PRENORM to *N*. Then

(1) The procedure terminates and outputs an acyclic *X*-network $\text {Prenorm}(N)$ which is pre-normal.

(2) $\text {Prenorm}(N)$ depends only on the geometry of *N*.

(3) The projection $\psi :N\rightarrow \text {Prenorm}(N)$ is a leaf-preserving CSD map.

(4) Let $E_1 =\{(u,v)\in A: \psi (u)\ne \psi (v)\}$. Then $(\psi ^{-1},E_1)$ is a wired lift of $\text {Prenorm}(N)$ into *N* that contains no dashed arcs.

### Proof

If the procedure returns *N* and $r=0$, then (1) is immediate. Otherwise, by Theorem 4.4, $N_1=SCD(N)$ is an acyclic *X*-network which contains no trivial vertices. If it contains no obstacles, it is pre-normal by Theorem 7.1, $r=1$, and (1) follows. Otherwise, it contains at least one obstacle *c*. For each obstacle *c* of type *k* and each allowable *k*-fold parent chain $p^k, \cdots , c$ for *c* the set $\{(p^k, p^{k-1}), \cdots , (p^1, c)\}$ contains no *X*-arc since the obstacle *c* cannot be a leaf. Hence *D*(*c*) contains no *X*-arc, so *D* contains no *X*-arc. By Theorem 3.8, $N_2=M_D(N_1)$ is an acyclic *X*-network, which we expect will be pre-normal by Lemmas 7.3 and 7.4. If $N_2$ contains no obstacles, it is pre-normal by Theorem 7.1, and $\text {Prenorm}(N)=N_2$, so (1) is true. Otherwise it contains an obstacle and the procedure returns to step 5.

Each time step 5 is utilized on $N_i$, the corresponding set *D* is nonempty, so more arcs are contracted and $N_{i+1}$ contains fewer vertices and fewer arcs. Since *N* is finite, the procedure must terminate. This proves (1).

The procedure never involves a choice, such as favoring some obstacles or some allowable parent chains over others. Hence (2) is true.

We wish to see (3). If $r=0$ then $\text {Prenorm}(N)=N$ and $\psi$ is the identity map. Otherwise, let $\psi _1:N\rightarrow \text {SCD}(N)=N_1$ be the projection from Theorem 4.4. If $r=1$ then $\text {Prenorm}(N)=N_1$ and $\psi _1: N\rightarrow \text {Prenorm}(N)$ proves (3). Suppose $r>1$. For $1<i\le r$ let $\psi _i: N_{i-1}\rightarrow M_D(N_{i-1}) = N_i$ be the projection. Then $\psi :N\rightarrow N_r=\text {Prenorm}(N)$ is the composition $\psi =\psi _r \circ \psi _{r-1} \circ \cdots \circ \psi _1$ and proves (3), since it is a composition of leaf-preserving CSD maps.

For (4), since $\psi$ is a CSD map, $(\psi ^{-1}, E_1)$ is a wired lift by Theorem 6.1(1). It has no dashed arcs since redundant arcs are allowed in $\text {Prenorm}(N)$. $\square$

Since $\text {Prenorm}(N)$ is a pre-normal acyclic *X*-network, we remove the redundant arcs to form $\text {R}(\text {Prenorm}(N))$, which will be normal. It may, however, contain trivial vertices, so we define $\text {Norm}(N)=\text {T}(\text {R}(\text {Prenorm}(N)))$, which will be normal and contain no trivial vertices. We call $\text {Norm}(N)$ the *normalization* of *N*. The next theorem records its basic properties.

### Theorem 7.8

Suppose *N* is an *X*-network. Let $\psi _1:N\rightarrow \text {Prenorm}(N)$ be the projection CSD map. Then

(1) $Norm(N)=\text {T}(\text {R}(\text {Prenorm}(N)))$ is a normal acyclic *X*-network containing no trivial vertices, hence a phylogenetic *X*-network.

(2) The definition of $\text {Norm}(N)$ depends only on the geometry of *N*.

(3) The projection $\psi _2:\text {R}(\text {Prenorm}(N))\rightarrow \text {Norm}(N)$ is a leaf-preserving CSD map.

(4) The composition $f=\psi _2 \circ \psi _1$ as maps of vertices from *N* to $\text {Norm}(N)$ is a connected map.

(5) There is a wired lift of $\text {Norm}(N)$ into *N* which may contain dashed arcs.

(6) $$Cl(\text {Norm}(N)) = Cl(\text {Prenorm}(N))$$.

### Proof

For (1) note $\text {R}(\text {Prenorm}(N))$ is an acyclic *X*-network by Theorem 5.1 which is normal by Theorem 7.1. Hence (1) follows from Theorem 7.6. Moreover, (2) is obvious since $\text {Prenorm}(N)$ depends only on the geometry of *N*. Then (3) follows from Theorem 7.6. Next (4) and (5) follow from Theorem 6.4. Finally (6) follows from Theorems 5.2 and 7.6. $\square$

### Remark

There is an interesting variant of the procedure PRENORM. Define the procedure VARIANT PRENORM to be exactly like PRENORM except that step (5c) is replaced by

(5$c'$) Select exactly one allowable *k*-fold parent chain $p^k, \cdots , c$ for *c*, and let $D(c) = Dc(p^k, \cdots , c)= \{(p^k, p^{k-1}), \cdots , (p^1, c)\}$.

We will abbreviate the name of the procedure to VARIANT. Thus while PRENORM uses *all* allowable *k*-fold parent chains for each obstacle *c* of type *k*, VARIANT would use just *one* allowable parent chain for each such obstacle. The following theorem shows that the output of VARIANT has interesting properties. The proof is like those of Theorems 7.7 and 7.8 and is omitted.

### Theorem 7.9

Let $N=(V,A,\rho ,\phi )$ be an *X*-network.

Apply procedure VARIANT PRENORM to *N*. Then

(1) The procedure terminates and outputs an acyclic *X*-network $N_r$ which is pre-normal.

(2) The projection $\psi _1:N\rightarrow N_r$ is a leaf-preserving CSD map.

(3) There is a wired lift of $N_r$ into *N* that contains no dashed arcs.

Let $$M_r= \text {T}(\text {R}(N_r))$$ Then

(4) $M_r$ is a normal acyclic *X*-network containing no trivial vertices, hence a phylogenetic *X*-network.

(5) The projection $\psi _2:\text {R}(N_r)\rightarrow M_r$ is a leaf-preserving CSD map.

(6) The composition $\psi _2 \circ \psi _1: N \rightarrow M_r$ as maps of vertices is a connected map.

(7) There is a wired lift of $M_r$ into *N*.

The output $N_r$ of VARIANT is called *a variant prenormalization* of *N* and is denoted $\text {Prenorm}_{{V,C}}(N)$ or for simplicity $\text {Prenorm}_V(N)$. Here *C* records the particular choice of allowable *k*-fold parent chain for *c* made in step (5$c'$) each time there was more than one allowable *k*-fold parent chain for *c* to choose among. Similarly $M_r= \text {T}(\text {R}(\text {Prenorm}_V(N)))$ is called *a variant normalization* of *N* and is denoted $\text {Norm}_{V,C}(N)$ or $\text {Norm}_V(N)$. Note that $\text {Norm}_V(N)$ and $\text {Prenorm}_V(N)$ will not necessarily depend only on the geometry of *N*; instead, the result will depend on all the choices *C* of the parent chains when there is more than one possible. In certain circumstances, however, it might be preferable. For example, the researcher might have additional information suggesting that the relevant gene flow is much more likely along one parent chain than another, in which case the least probable parent chain should be selected for merging.

It frequently happens that $\text {Prenorm}_V(N)$ has more vertices and hence higher resolution than $\text {Prenorm}(N)$. The following lemma indicates one source of this inequality. To obtain a short proof, we assume very strong hypotheses.

### Lemma 7.10

Suppose $N=(V,A,\rho ,\phi )$ is an *X*-network. Assume when $i=1$ that in step 6 of PRENORM the merging set of arcs in $N_1=(V_1, A_1,\rho _1,\phi _1)$ is *D*, while in step 6 of VARIANT the merging set is *E*. Then

(1) $$E\subseteq D$$.

(2) If for some obstacle *c* of type *k* there is more than one allowable *k*-fold parent chain, then $E\subsetneq D$.

(3) If *u* and *v* are vertices of $V_1$ and $u\sim _E v$, then $u\sim _D v$.

(4) $$K(E)\subseteq K(D)$$.

(5) If *u* and *v* are vertices of $V_1$ and $u\sim _{K(E)} v$, then $u\sim _{K(D)} v$.

(6) If $K(E)\subsetneq K(D)$, then $M_E(N_1)$ has strictly more vertices than $M_D(N_1)$.

(7) If $K(E)\subsetneq K(D)$ and $r=2$ for both PRENORM and VARIANT, then $\text {Prenorm}_V(N)$ has strictly more vertices than $\text {Prenorm}(N)$.

### Proof

(1) is true since for a given obstacle *c* of type *k*, *E* contains *exactly one* allowable *k*-fold parent chain for *c*, while *D* contains *all* allowable *k*-fold parent chains for *c*. Then (2) is obvious and (3) follows from (1). For (4), go through the proof of Theorem 3.7 and note that every time an arc is added to make *K*(*E*), it necessarily must be added also to *K*(*D*). Then (5) follows from (4). For (6), the vertices of $M_D(N_1)$ are the equivalence classes of $V(N_1)$ under $\sim _{K(D)}$, while the vertices of $M_E(N_1)$ are the equivalence classes of $V(N_1)$ under $\sim _{K(E)}$. Since $K(E)\subsetneq K(D)$, (6) follows. Now (7) is clear. $\square$

A calculation of $\text {Norm}_V(N)$ is illustrated below in Example 7.

Other variants making other choices of parent chains are possible as well.

### Example 3

This example continues Example 1 in Sect. 4. Consider the network *N* in Fig. 4. The first step is to compute $\text {SCD}(N)$, shown in Fig. 5. Since $\text {SCD}(N)$ is a tree, it has no obstacle, the height $r= 1$ and $\text {Prenorm}(N) = \text {SCD}(N)$. Since there are no redundant arcs, we find $\text {Norm}(N) = \text {T}(\text {SCD}(N))=\text {SCD}(N)$. Moreover, $d_{RF}(N,\text {Norm}(N))=0$. It is easy to see in this example that $\text {FHS}(N)$ is the star tree consisting of the root 7 together with an arc from 7 to each of the six leaves. Moreover $\text {SCD}(N)$ (and therefore *N* has four more distinct clusters than the tree $\text {FHS}(N)$. Hence $d_{RF}(N,\text {FHS}(N))=4$. In this example, $\text {Norm}(N)$ outperforms $\text {FHS}(N)$.

**Fig. 9:**
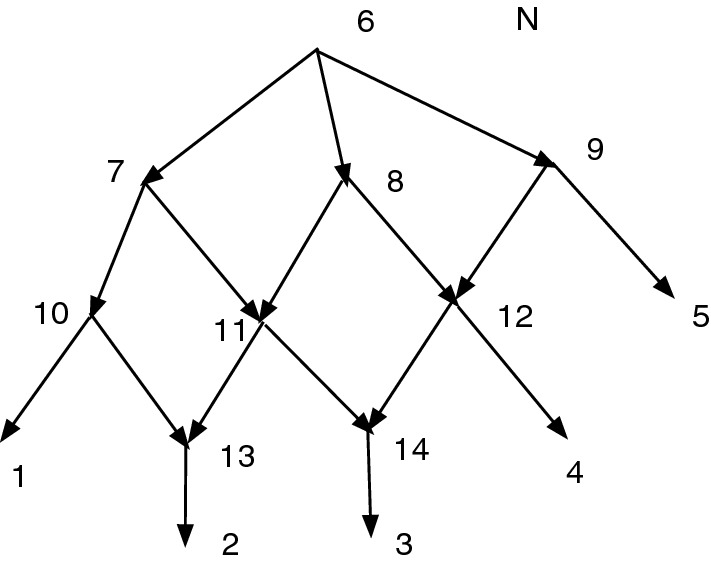
An SCD *X*-network *N*

**Fig. 10:**
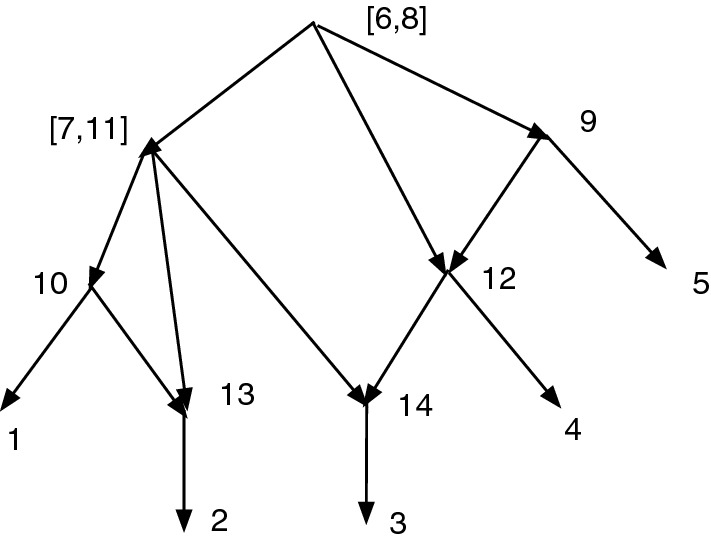
$M_D(N)$ for *N* in Fig. 9, where $D = \{(7,11), (6,8)\}$. Note that $\text {Prenorm}(N)=M_D(N)$ and the normalization is obtained by removing the redundant arcs ([7,11],13) and ([6,8],12) and then the resulting trivial vertex 13

**Fig. 11:**
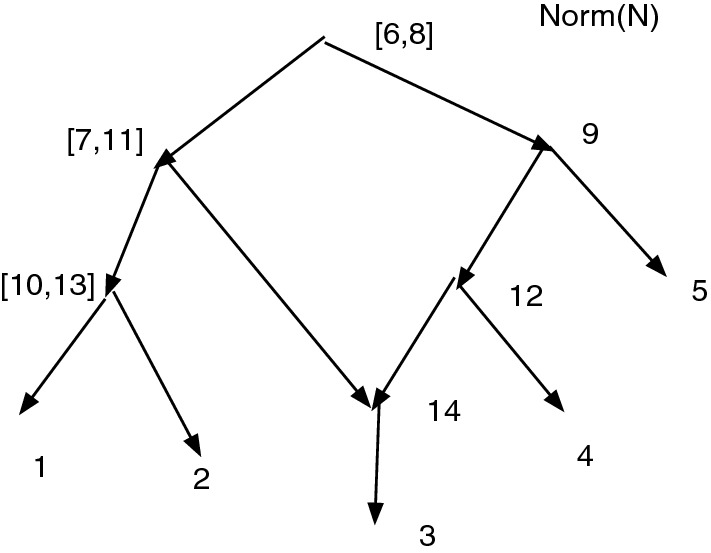
$\text {Norm}(N)=\text {T}(\text {R}(M_D(N)))$ for *N* in Fig. 9, where $D = \{(7,11), (6,8)\}$. Note the removal of the trivial vertex 13 from Fig. 10

**Fig. 12:**
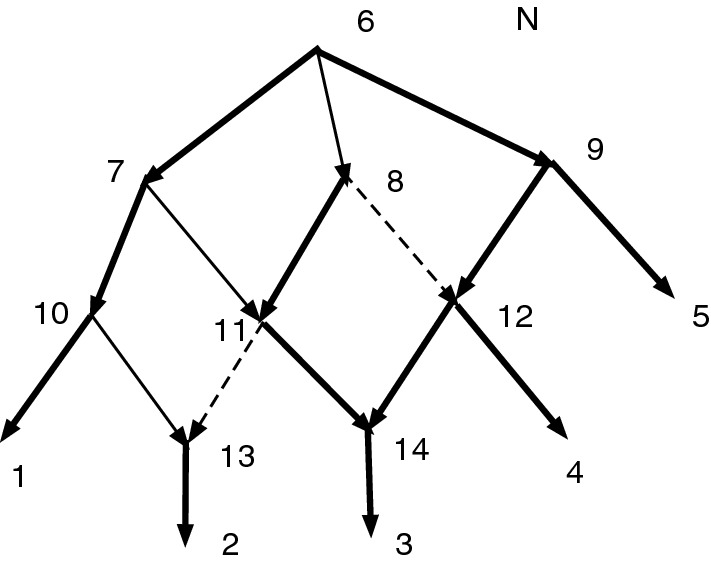
Figure 9 redrawn to exhibit the wired lift of $\text {Norm}(N)$ into *N*. Thin solid arcs are merged in $\text {Norm}(N)$. Dashed arcs give rise to redundant arcs of $M_D(N)$ and are not included in $E_1$. Wide solid arcs are in $E_1$

### Example 4

Consider *N* in Fig. 9. Let $N_0=N$, so $N_1=\text {SCD}(N)=N$ since *N* is already SCD. $N_1$ has two obstacles: 8 and 11. Obstacle 8 is type 1 with one allowable parent chain 6,8 and $D(8) = \{(6,8)\}$. Obstacle 11 has type 1 with allowable chain 7,11 and $D(11) = \{(7,11)\}$. (Note that 8,11 is not an allowable chain since 12 is hybrid.) Hence $D = D(8) \cup D(11)$ $= \{(6,8), (7,11)\}$.

*D* is strongly closed, and we find $N_2=M_D(N)=Q_D(N)$, shown in Fig. 10 with redundant arcs ([7,11],13) and ([6,8],12). There are no obstacles, so the height $r=2$ and $\text {Prenorm}(N)=N_2$. We remove the redundant arcs to find $\text {R}(M_D(N))$, which contains the trivial vertex 13. Then we compute $\text {T}(\text {R}(M_D(N)))$ to remove the trivial vertex by merging the arc (10,13) as in Theorem 7.6 to yield $\text {Norm}(N)=\text {T}(\text {R}(M_D(N)))$, shown in Fig. 11.

The projection map $\psi : N \rightarrow M_D(N)$ has $\psi (7) = \psi (11)$ $= [7,11]$ and $\psi (6) = \psi (8)=[6,8]$. For other vertices *v*, $\psi (v) = v$. Without the redundant arc ([7,11],13), $\psi$ would not be a CSD map since *N* contains the arc (11,13).

Let $\psi _2:R(M_D(N))\rightarrow \text {Norm}(N)$ be the projection and $f=\psi _2\circ \psi : N\rightarrow \text {Norm}(N)$ be the composition of the vertex maps. Note *f* is a connected map. Figure 12 shows Fig. 9 redrawn to exhibit the wired lift $(f^{-1},E_1)$ of $\text {Norm}(N)$ into *N*. The thin solid arcs show immediately that there were identifications $6\sim 8$, $7\sim 11$, and $10\sim 13$. The dashed arcs correspond to the redundant arcs ([7,11],13) and ([6,8],12) in Fig. 10, which are not present in $\text {Norm}(N)$. We see, for example, there is a unique g-path from 8 to 2, given by 8,6,7,10,13,2. Hence in $\text {Norm}(N)$ there is a path from $\psi (8)=[6,8]$ to $\psi (2)=2$.

It is easy to compute that $d_{RF}(N,\text {Norm}(N)) = d_{RF}(N, \text {T}(\text {R}(M_D(N))))$ $=d_{RF}(N,\text {R}(M_D(N)))$ $=d_{RF}(N, M_D(N))=2$ using Theorems 7.6 and 5.2. We identify the two relevant clusters by noticing that *N* contains vertices with clusters $\{1,2,3\}$ and $\{2,3\}$ that are not in $M_D(N)$, while every cluster of $M_D(N)$ and hence of $\text {R}(M_D(N))$ is also a cluster of *N*. In this example, $\text {FHS}(N)=\text {Norm}(N)$.

**Fig. 13:**
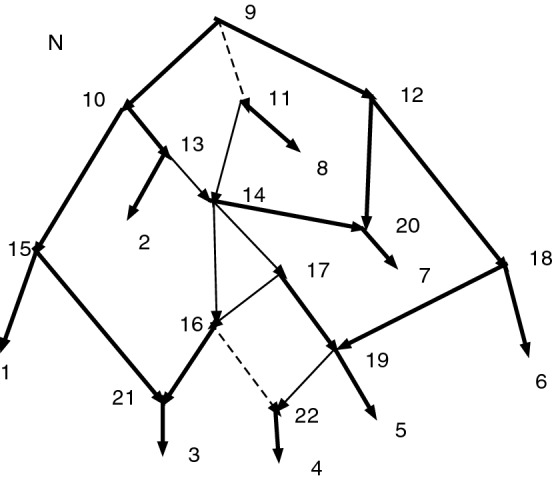
The wired lift of $\text {Norm}(N)$ for an *X*-network *N* with a single obstacle 16 of type 3. *N* is seen if all arcs are instead made wide solid. The wired lift of $\text {Prenorm}(N)$ is seen if the arcs (9,11), (16,22), and (19,22) are all instead made wide solid

**Fig. 14:**
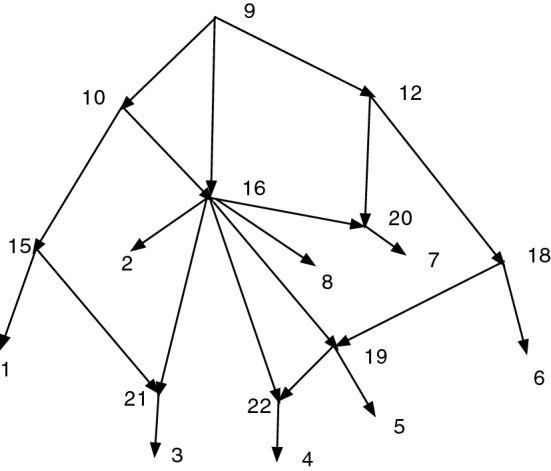
$\text {Prenorm}(N) = M_D(N)$ for the *N* of Fig. 13. The vertex [11, 13, 14, 16, 17] is labeled 16. The arcs (9,16) and (16,22) are redundant. $\text {Norm}(N)$ is found by removing the two redundant arcs and then using $\text {T}$ to remove the resulting trivial vertex 22 by merging (19,22)

### Example 5

Figure 13 shows the wired lift of $\text {Norm}(N)$ into an *X*-network *N* with a single obstacle 16 of type 3. *N* is seen by changing all thin solid or dashed arcs to wide solid. *N* is easily verified to be already SCD, so $N_1=N$. One 3-fold parent chain in *N* is 11, 14, 17, 16; another is 13, 14, 17, 16. The chain 13,14,16 is not an allowable parent chain because (14,16) is redundant. To find $\text {Prenorm}(N)$, use $D = \{(11,14),(14,17),(17,16)$, $(13,14)\}$.

$$ M_D(N)$$ is shown in Fig. 14. Note $$M_D(N)$$ has no obstacles, hence is pre-normal. Thus $$\text {Prenorm}(N) = M_D(N)$$ and the height $$r=2$$. The point [11,13,14,16,17] is labeled 16. If $$\psi : N \rightarrow M_D(N)$$ is the projection, then $$\psi ^{-1}(\psi (16)) = \{11, 13, 14, 16$$,$$17\}$$. $$M_D(N)$$ contains redundant arcs (9, 16) and (16, 22), arising from the arcs (9,11) and (16,22) in *N*. When these arcs are removed from $$M_D(N)$$ and the resulting trivial vertex 22 is removed, we obtain $$\text {Norm}(N) = \text {T}(\text {R}(M_D(N)))$$.

As maps of vertex sets $\psi _1:N\rightarrow M_D(N)$ and $\psi _2: \text {R}(M_D(N))\rightarrow \text {Norm}(N)$ can be composed to yield the resulting connected map $f=\psi _2\circ \psi _1:N\rightarrow Norm(N)$; it is not a CSD map because the vertex map from $M_D(N)$ to $R(M_D(N))$ is not CSD. The wired lift $(f^{-1},E_1)$ of $\text {Norm}(N)$ is shown in Fig. 13. The arcs (9,11) and (16,22) are dashed because they are pre-arcs to the redundant arcs of $M_D(N)$, which are not arcs of $\text {Norm}(N)$, hence are not in $E_1$ of the wired lift, using Theorem 6.3. Arcs (*u*, *v*) such that $f(u)=f(v)$ are thin solid. Hence arcs in the induced subgraph of $f^{-1}(f(16))= \{11, 13, 14, 16$,$17\}$ are thin solid. The arc (19, 22) is thin solid because it was merged to remove the trivial vertex 22. Note the g-path 16,17,19,22,4 from 16 to 4; but 16,22,4 is not a g-path.

The wired lift of $\text {Prenorm}(N)$ would be seen in Fig. 13 if the arcs (9,11), (16,22), and (19,22) are made wide solid. The first two would be wide since in $\text {Prenorm}(N)$ both (9,16) and (16,22) are arcs. Arc (19,22) would be wide since 22 is not a trivial vertex in $\text {Prenorm}(N)$ and is not removed.

## Examples with Real Data

This section contains two examples from real biological data.

### Example 6

**Fig. 15:**
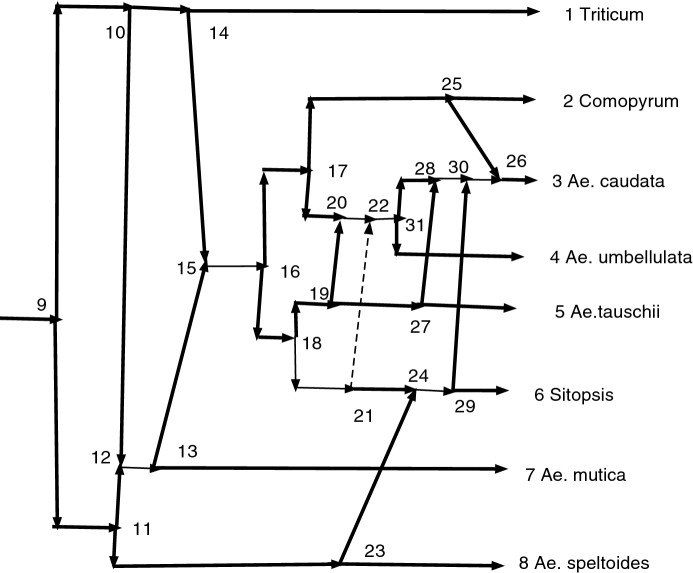
The wired lift of $\text {Norm}(N)$ for the diploid Aegilops/Triticum species in Glémin et al. (2019). Wide arcs are in $E_1$. Thin solid arcs represent identifications; thus $20\sim 22\sim 31$, $15\sim 16$, $12\sim 13$, $18\sim 21$, $24\sim 29$, and $28\sim 30\sim 26$. The dashed arc (21,22) corresponds to a redundant arc in $\text {Prenorm}(N)$ and may not be used for g-paths. If all arcs are instead wide solid, we obtain *N*

Glémin et al. (2019) study pervasive hybridizations of wheat relatives. Their Fig. 5 shows their proposed scenario for the history of diploid Aegilops/Triticum species. Let *N* be their graph. A wired lift of $\text {Norm}(N)$ is shown in our Fig. 15. The network *N* is seen if each arc in Fig. 15 is made wide solid. In $\text {SCD}(N)$ we find only a single pre-normal obstacle 21 of type 1. The height of the computation is $r=2$. When we compute $\text {Prenorm}(N)$, there is a single redundant arc. $\text {Norm}(N)$ contains 23 vertices and 29 arcs; it is thus simpler than *N*, which contains 31 vertices (of which eight are hybrid), and 38 arcs. We find $d_{RF}(N,\text {Norm}(N))=1$. It is interesting that our dashed arc (21,22) is also dashed in Glémin et al. (2019) to indicate a less likely event. It turns out that in this case $\text {FHS}(N)\cong \text {Norm}(N)$.

**Fig. 16:**
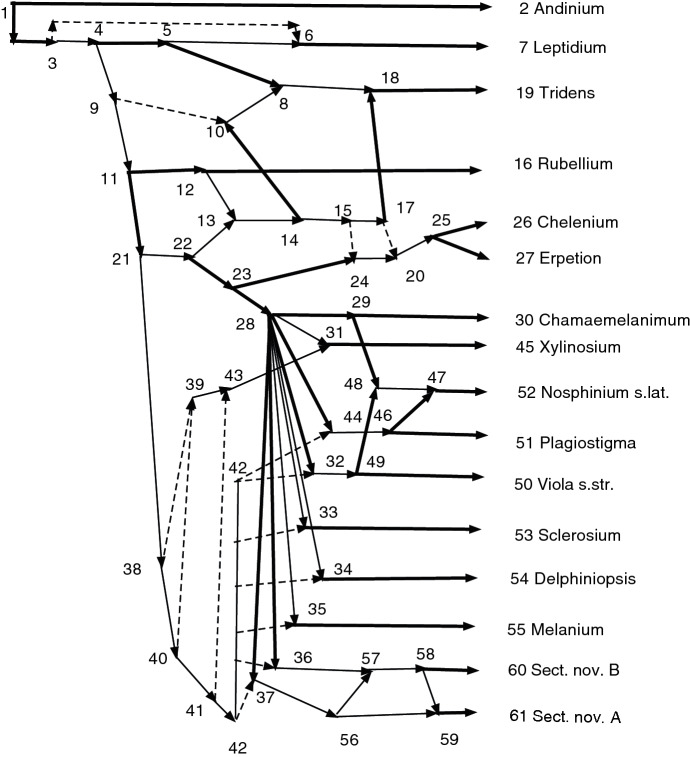
The wired lift of $\text {Norm}(N)$ for the *Viola* data *N* in Marcussen et al. (2015). The entire vertical line labeled 42 represents one vertex with out-degree 7

### Example 7

Marcussen et al. (2015) exhibit a network *N* for the angiosperm genus *Viola* in their Fig. 4. Our methods find $N_1=\text {SCD}(N)$ has 2 obstacles. One obstacle is type 1 with two allowable 1-fold parent chains. The other is type 2 with one allowable 2-fold parent chain. Thus for computing $\text {Prenorm}(N)$, *D* contains 4 arcs. $M_D(N_1)$ has no obstacles, so $\text {Prenorm}(N)=M_D(N_1)$ and the height is two. A wired lift of $\text {Norm}(N)=\text {T}(\text {R}(\text {Prenorm}(N)))$ is shown in Fig. 16. We see that $\text {Norm}(N)$ has 29 vertices (equivalence classes under thin solid arcs). It turns out to have 31 arcs, while the wired lift has 34 wide solid arcs. If $\psi :N\rightarrow \text {Norm}(N)$ is the connected map, more than one wide solid arc (*u*, *v*) can map to the same arc $(\psi (u),\psi (v))$ of $\text {Norm}(N)$. Thus (14,10) and (17,18) map to the same arc in $\text {Norm}(N)$, as do (28,37) and (28,36), and also (11,12) and (11, 21).

$\text {FHS}(N)$ is drawn in Francis et al. (2021) in their Fig. 3. We compute that $d_{RF}(N,\text {FHS}(N)) = 4$ while $d_{RF}(N,\text {Norm}(N)) = 5$. Thus $\text {FHS}(N)$ is a better approximation to *N* than $\text {Norm}(N)$ but lacks a wired lift.

If we use instead VARIANT PRENORM, there are two possible normal networks $\text {Norm}_V(N)$ that can result, depending on the choice of the 1-fold parent chain for the type 1 obstacle [13,14,15,17] in $\text {SCD}(N)$. One allowed parent chain is 12, [13,14,15,17]; the other is [21,22], [13,14,15,17]. Thus for computing $\text {Prenorm}_V(N)$, the set *D* has 3 rather than 4 arcs, leading to one more vertex in $\text {Prenorm}_V(N)$ compared to $\text {Prenorm}(N)$. For both networks $\text {Norm}_V(N)$, $d_{RF}(N,\text {Norm}_V(N))=4$, so they both approximate *N* as well as does $\text {FHS}(N)$ but depend on the choice of 1-fold parent chain.

Both such $\text {Norm}_V(N)$ have wired lifts into *N* by Theorem 7.9. The wired lift of each $\text {Norm}_V(N)$ is very similar to that for $\text {Norm}(N)$ with one additional wide solid arc replacing a thin solid arc. For one $\text {Norm}_V(N)$ the wired lift is given by making the arc (12,13) in Fig. 16 be wide solid; for the other $\text {Norm}_V(N)$, the only change is that the arc (22,13) in Fig. 16 is wide solid.

Both variant normalizations satisfy $d_{RF}(\text {Norm}_V(N),\text {FHS}(N))=2$, so neither agrees with $\text {FHS}(N)$.

Further comments concerning this example are given in Sect. 9.

## Discussion

**Comparison of** $\text {Norm}(N)$ **and** $\text {FHS}(N)$ Let $N=(V,A,\rho ,\phi )$ be an *X*-network. It is interesting to contrast $\text {Norm}(N)$ with $\text {FHS}(N)$, defined in Francis et al. (2021). Both are uniquely determined normal phylogenetic *X*-networks depending only on the geometry of *N*. Both allow vertices of *N* to have in-degree greater than 2 or out-degree greater than 2, and both apply quite generally.

$\text {FHS}(N)$ is fast to compute using Huson and Steel (2020) and very elegant. It works by locating the “visible” vertices of *N*. A vertex *v* is *visible* if there exists $x\in X$ such that every path from $\rho$ to $\phi (x)$ contains *v*. This set of visible vertices forms the initial vertex set of $\text {FHS}(N)$. Hence each initial vertex of $\text {FHS}(N)$ can be highlighted in the diagram for *N*, as is done in Francis et al. (2021). At the end, trivial initial vertices of $\text {FHS}(N)$ are suppressed. In a tangled network like our Fig. 4, the only visible vertices are the root and the leaves, since there is a great multiplicity of possible paths from the root to a given leaf. In such a situation, $\text {FHS}(N)$ does not perform well. For less tangled networks such as Example 7 the computation works well. Perhaps it would be useful in general to compute $\text {FHS}(\text {SCD}(N))$.

The arcs of $\text {FHS}(N)$ are harder to interpret than the vertices. In $\text {FHS}(N)$ there is an arc (*u*, *v*), where *u* and *v* are distinct visible vertices of *N*, precisely when $u\le v$ in *N* and there is no third visible vertex *w* such that $u\le w$ and $w\le v$. Thus, for example, two different arcs $(u,v_1)$ and $(u,v_2)$ emerging from the same *u* could be present because of directed paths in *N* from *u* to $v_1$ and from *u* to $v_2$ such that the paths have significant overlap, invisible in $\text {FHS}(N)$.

Consider again Fig. 16 where *N* is for the *Viola* genus of Marcussen et al. (2015). The diagram of *N* is exactly Fig. 16 in which all arcs are drawn wide solid. The vertices of $\text {FHS}(N)$, before suppression of the trivial vertices, are the 43 visible vertices out of the 61 vertices of *N*. None of the vertices 13, 14, 15, 17 of *N* (those relevant to the obstacle [13,14,15,17] crucial to our discussion of the VARIANT calculation) are visible and hence they do not appear in $\text {FHS}(N)$. $\text {FHS}(N)$ has arc (22,18) because (a) 22 and 18 are visible, (b) 22, 13, 14, 10, 8, 18 is a path in *N*, and (c) there is no other path from 22 to 18 containing a third visible vertex. Similarly $\text {FHS}(N)$ has arc (12,20) because of the path 12, 13, 14, 15, 17, 20, and it has arc (12,18) because of the path 12, 13, 14, 10, 8, 18 in *N*. Thus these three distinct arcs in $\text {FHS}(N)$ arise from overlapping paths in *N* involving 13 and 14.

In contrast, the arcs of $\text {Norm}(N)$ are easy to interpret. The wide solid arcs highlight the arcs *N* that appear in $\text {Norm}(N)$; the thin dashed arcs indicate redundant arcs in $\text {Prenorm}(N)$ and must be avoided in *g*-paths; the thin solid arcs tell what arcs must be merged to obtain the normal network $\text {Norm}(N)$. The use of *g*-paths lets us understand $\text {Norm}(N)$ from just the wired lift.

**Software** The author has written software using *Xcode* which implements the calculation of $\text {Norm}(N)$ somewhat interactively. It was essential for the examples based on real data. It computes $\text {SCD}(N)$, $M_D(N)$, $\text {R}(N)$, and $\text {T}(N)$ and locates all obstacles. It finds all allowable 1-fold and 2-fold parent chains, but obstacles of type $k\ge 3$ must be handled interactively. The software is far from ready for general use, but it shows that the calculations can be automated.

**Future work** One can ask whether there are other classes of networks besides normal networks for which a similar construction could be used to simplify a network *N* into one of this other class. Suppose, given an *X*-network *N*, we sought a tree-child *X*-network *C*. Since a tree child network may contain redundant arcs, we should like a construction that depends only on the geometry of *N* and yields a CSD map $\psi :N\rightarrow C$. At first glance we might think that we could use the CSD map $\psi :N\rightarrow \text {Prenorm}(N)$; but while $\text {Norm}(N)$ is tree-child, $\text {Prenorm}(N)$ need not be tree-child. The author is currently looking at such problems for tree-child and some other classes of networks.
